# Diffuse midline glioma treated with epigenetic agent-based immunotherapy

**DOI:** 10.1038/s41392-022-01274-7

**Published:** 2023-01-20

**Authors:** Linkai Jing, Zhihong Qian, Qiang Gao, Rui Sun, Zili Zhen, Guihuai Wang, Xuejun Yang, Haitao Li, Tiannan Guo, Wei Zhang

**Affiliations:** 1 Department of Neurosurgery, Beijing Tsinghua Changgung Hospital, Tsinghua University, Beijing, People’s Republic of China; 2 Department of Basic Medical Sciences, School of Medicine, Tsinghua University, Beijing, People’s Republic of China; 3 School of Clinical Medicine, Tsinghua University, Beijing, People’s Republic of China; 4 Key Laboratory of Structural Biology of Zhejiang Province, School of Life Sciences, Westlake University, Zhejiang, People’s Republic of China; 5 Institute of Basic Medical Sciences, Westlake Institute for Advanced Study, Zhejiang, People’s Republic of China; 6 Ministry of Education Key Laboratory of Protein Sciences, Beijing Advanced Innovation Center for Structural Biology, Beijing Frontier Research Center for Biological Structure, Department of Basic Medical Sciences, School of Medicine, Tsinghua University, Beijing, People’s Republic of China; 7 Tsinghua-Peking Center for Life Sciences, Tsinghua University, Beijing, People’s Republic of China; 8 IDG/McGovern Institute for Brain Research, School of Life Sciences, Tsinghua University, Beijing, People’s Republic of China

**Keywords:** Cancer therapy, CNS cancer

**Dear Editor**,

Diffuse midline glioma, H3K27-altered (DMG), is a pediatric-type high-grade diffuse glioma that preferentially localizes to the brainstem or pons, thalamus, and spinal cord. Surgical resection is challenging and biopsy is often performed in most cases. To date, no conventional, targeted or immune therapy has been convincingly shown to improve patients’ overall survival (OS). Effective treatment guideline is absent and remains to be established. Prognosis of DMG patients is poor and 2-year survival rate is <10%. Noteworthily, DMG arising from spinal cord with leptomeningeal metastasis (LM) is rarely reported. Historically, LM of high-grade gliomas have largely been excluded from clinical trials owing to a particularly poor prognosis, with a median OS of 1.6–3.8 months.^1^

As a classical “oncohistone”, H3K27M––lysine to methionine substitution at position 27 in histone 3––has driven epigenomic reprogramming in DMG. DMG exhibits a distinct chromatin landscape characterized by global loss of H3K27 trimethylation (H3K27me3) and gain of H3K27 acetylation (H3K27ac). H3K27ac is enriched in intergenic regions, leading to increased expression of endogenous retroviral elements that can be further enhanced by epigenetic drugs. This viral mimicry can prime DMG for innate immune responses and promote immune cell activation. Building on the premise, epigenetic therapy is envisaged to augment anti-tumor immunity and convert DMG from immune “cold” to immune “hot”. Unfortunately, neither epigenetic nor its combinatorial therapy has shown survival efficacy in treating glioma patients. Clinical trials combining epigenetic therapy with immune checkpoint blockade (ICB) in DMG patients is lacking. We present our experience of applying epigenetic agent-based immunotherapy in treating DMG patients.

Patient 1 was a 28-year-old man diagnosed with DMG in the thoracic cord on September 13th 2020 (Supplementary Table 1). He received tumor resection without any adjuvant therapy at initial diagnosis and recovered with minor neurological deficits. Three months after the surgery, he developed leg weakness, hiccups, and lethargy. His neurological symptoms progressively worsened. On January 11th, 2021 (Day 1), the patient was admitted to our hospital with confusion and a suddenly deteriorating Glasgow Coma Scale score of 8/15. Magnetic resonance imaging (MRI) showed evidence of thoracic tumor recurrence and LM involving brain (Fig. 1a, Day 2). We performed subtotal resection of the thoracic lesion and biopsy of LM in dorsal medulla on Day 5. Pathologic diagnosis confirmed DMG (World Health Organization grade 4) in both locations (Supplementary Fig. 1). Therapeutic details are described in Supplementary methods and Supplementary Fig. 2, and treatment related adverse events are shown in Supplementary Table 2. Responses of patient-derived tumor cells to therapy in in vitro assays are presented in Supplementary Figs. 3 and 4. During treatment, all lesions continued to regress until the data cut-off date (Day 371), with those in the suprasellar region (T1) and dorsal medulla (T2) measured 137 and 14 mm^3^, corresponding to >94% reduction of baseline volume (Supplementary Table 3). Leptomeningeal and periventricular enhancement were no longer detectable on Day 367 (Fig. 1a and Supplementary Figs. 5–8). His quality of life improved dramatically through the treatment and he went back to normal life activities since cycle 11 (Supplementary Fig. 9 and Table 4).

**Fig. 1:**
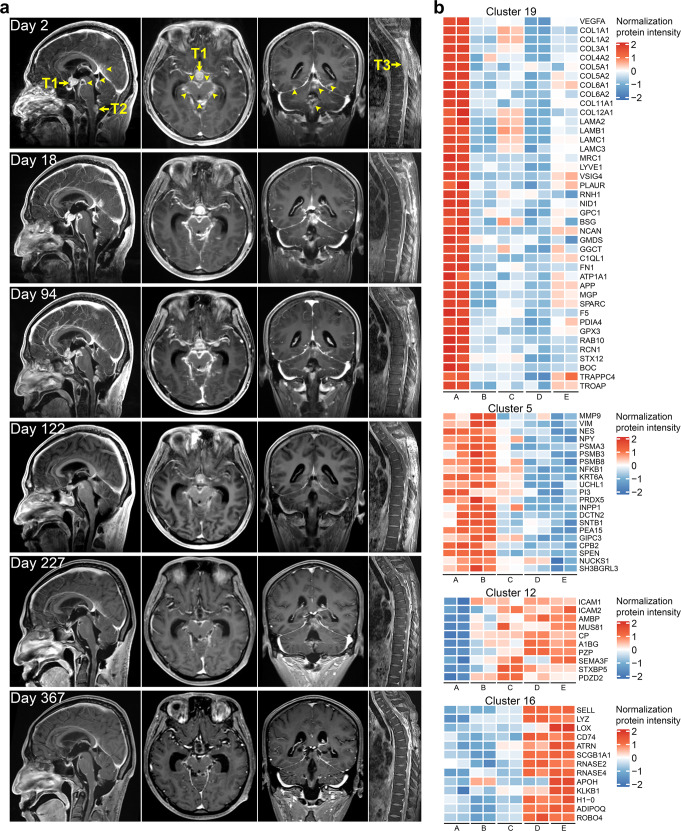
Therapeutic response. **a** Contrast-enhanced T1-weighted MRI images. Images were obtained on Day 2, 18, 94, 122, 227, and 367. Baseline MRI (Day 2) showed LM in the suprasellar region (T1) and dorsal medulla (T2) and recurrence of the thoracic tumor (T3). Foci of linear leptomeningeal and periventricular enhancement are highlighted with arrowheads. Notably, in cycle 1, his consciousness recovered to drowsiness after thoracic tumor resection, but 10 days later he developed diabetes insipidus and hyponatremia due to increased volume of the suprasellar nodule from a baseline of 1905 to 2606 mm^3^ (see Supplementary Fig. 2a and Table 3 for details). Leptomeningeal and periventricular enhancement also progressed. The electrolyte and fluid imbalance were transient and resolved within 2 cycles of treatment. His consciousness fully recovered since cycle 3 with concurrent improvement of global neurocognitive function (See Supplementary Fig. 9 and Table 4 for details). When radiotherapy was completed in cycle 6, the volume of all lesions had remarkably diminished, from a baseline of 1905 mm^3^ for T1 and 829 mm^3^ for T2, to 1615 and 265 mm^3^ on Day 122, respectively. Interestingly, in cycle 11 (Day 227), without any neurological symptom, the involvement of periventricular enhancement was slightly increased due to the reduced dose of panobinostat within this cycle, but decreased after dose increase in cycle 12 (Day 256) (see Supplementary Figs. 2a, 5–7 and Table 3 for details). **b** Differentially expressed proteins in CSF through the course of therapy. CSF proteins differentially expressed through the treatment course (ANOVA adjusted *p* < 0.05) were grouped into 20 clusters based on expression profiles (see Supplementary Fig. 10 for cluster characteristics). Heatmaps illustrating the relative expression of selected proteins that had a consistent trend in protein expression in CSF samples collected at various time points during treatment are shown. Refer to Supplementary Fig. 11 for the full list of proteins in each of the four clusters. Time points A–E represented Day 26, 72, 169, 321, and 340, respectively

We collected cerebrospinal fluid (CSF) from patient 1 on Day 26, 72, 169, 321, and 340 during follow-up, on which we further performed mass spectrometric analysis to profile the whole proteome. A total of 1812 proteins were identified, of which 1118 were differentially expressed across the time points tested and clustered into 20 groups based on expression patterns (Supplementary Figs. 10 and 11). We focused on four clusters of consistently changed proteins (Fig. 1b and Supplementary Table 5). Results indicated that tumor angiogenesis, metastasis, and proliferation-associated proteins such as VEGFA, collagen family, and laminin family were remarkably decreased since Day 26. Tumor invasion, migration, epithelial-mesenchymal transition, and stemness-related proteins such as MMP9, VIM and NES showed a steady decline since Day 72. Interestingly, a protease inhibitor, pregnancy zone protein (PZP), was increased since Day 26 and proteasome subunits (PSMA3, PSMB3, and PSMB8) were decreased since Day 72, suggesting protease inhibition may be involved in our treatment efficacy without using proteasome inhibitor such as marizomib, which was demonstrated as a promising therapeutic approach combined with panobinostat in DMG (Supplementary Table 6). MRC1, the marker of M2-like tumor-associated macrophages; VISG4, a negative regulator of cytotoxic T lymphocytes activation; PLAUR, a marker of immunosuppression in gliomas were decreased significantly from Day 26. With increase of immune cell migration and activation-associated proteins ICAM1 and ICAM2 since Day 72, immune activation and enhanced immune response-related proteins such as SELL, LYZ and ATRN were sharply increased from Day 169 to Day 321 and maintained in a high expression level till the last sampling day.

This patient’s tumor eventually recurred at two new locations after data cut-off. He survived for 20 months from diagnosis and 16 months from epigenetic agent-based immunotherapy. We further applied the therapy in 3 more DMG patients and all achieved significant clinical responses (Supplementary Figs. 12–14).

Nonmutational epigenetic reprogramming has been proposed as a new hallmark of cancer.^2^ However, epigenetic therapy alone has not achieved desired therapeutic response in glioma patients. Panobinostat was a histone deacetylase inhibitor (HDACi) approved for treating multiple myeloma. Although it did not significantly improve 6-month progression-free survival when combined with bevacizumab in patients with recurrent GBM and anaplastic glioma, experiments demonstrated it penetrated blood–brain barrier and is a promising agent in treating DMG (Supplementary Table 6). Compassionate use of panobinostat was well-tolerated in four DMG patients, one of which was spinal cord DMG with LM and the OS was 6 months.^3^ Similarly, we observed that panobinostat combined with bevacizumab had anti-tumor efficacy but was not sufficient to prevent progression of brain metastasis in an advanced spinal cord DMG patient (Supplementary Fig. 15). Tazemetostat, the first small molecule Enhancer of zeste homolog 2 inhibitor (EZH2i) for treating epithelioid sarcoma approved in 2020, has not ever been tested in glioma patients. Despite global loss of H3K27me3 in H3K27M cells, some tumor suppressor genes, such as CDKN2A, were retained epigenetically silent.^4^ Evidences from in vitro and in vivo studies support that EZH2i might be a specific anchored therapeutic approach for treating DMG through derepression of tumor suppressor genes^4^ and improvement of immunotherapeutic efficacy (Supplementary Table 6). Here, we provide the first study applying EZH2i as one of epigenetic agents in treating DMG patients.

Combined treatments of epigenetic agents with immunotherapy exhibited promising results in patients of various solid tumors and hematological malignancies, but not in gliomas (Supplementary Table 6). The overall benefit in patients with gliomas by using ICB remained dismal. PD-1 blockade did not provide a survival benefit for patients with GBM or hypermutated gliomas (Supplementary Table 6). In LM patients, single agent PD-1 inhibitor was well-tolerated, but the median OS remained poor at 3.6 months.^5^ Highly immunosuppressive tumor microenvironment and lack of tumor-infiltrating lymphocytes may account for the limited efficacy of ICB. In patient 1, proteomic data implicated anti-tumor effect and activated immune response in a time- and epigenetic treatment-dependent manner. To the best of our knowledge, this is the first report highlighting notable regression in advanced DMG patients with LM by using chromatin-targeting agent-based immunotherapy.

Collectively, our observations refreshed the paradigm that epigenetic therapy may contribute to reinvigorating the immunotherapeutic efficacy in immune “cold” gliomas. This could be leveraged to develop treatment strategies in the larger context of malignancies with epigenetic dysregulation.

## Supplementary information

Supplementary Materials

Fig S1

Fig S2

Fig S3

Fig S4

Fig S5

Fig S6

Fig S7

Fig S8

Fig S9

Fig S10

Fig S11

Fig S12

Fig S13

Fig S14

Fig S15

## Data Availability

The datasets generated during the current study are available from the corresponding author on reasonable request. Raw files of proteomics have been deposited to the iProX: IPX0005197001.
