# Supplementary figures and images for: Diffuse midline glioma treated with epigenetic agent-based immunotherapy
Source: Signal Transduct Target Ther. 2023 Jan 20;8:23. doi: 10.1038/s41392-022-01274-7 (PMC9852273; doi:10.1038/s41392-022-01274-7)

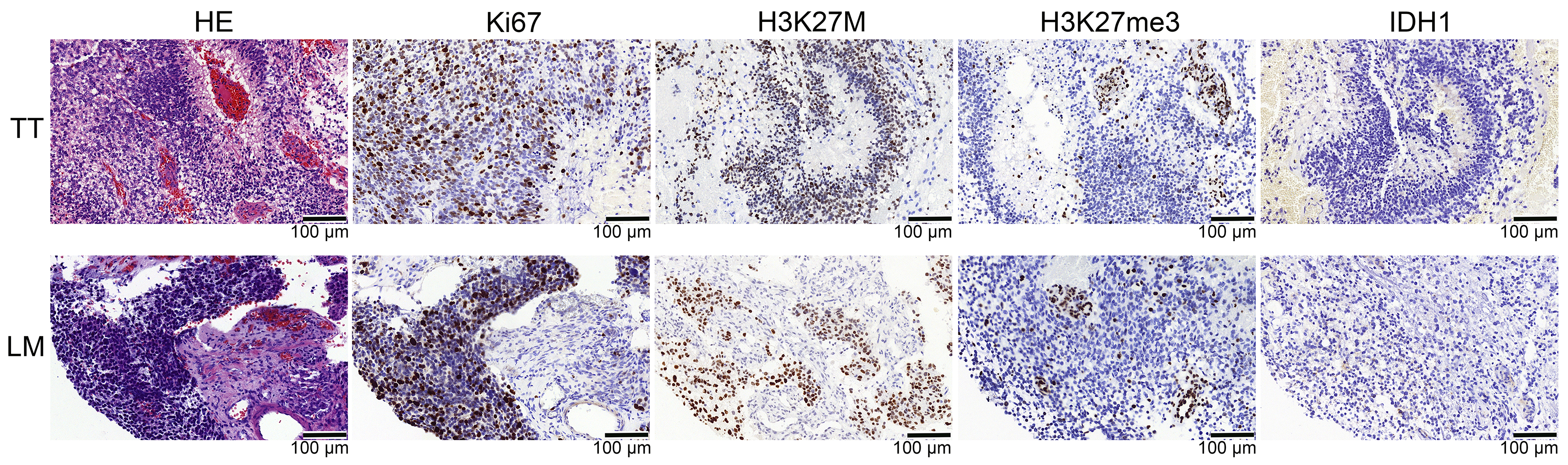

Supplement: Supplementary file 2 — Fig S1 [file 41392_2022_1274_MOESM2_ESM.tif]

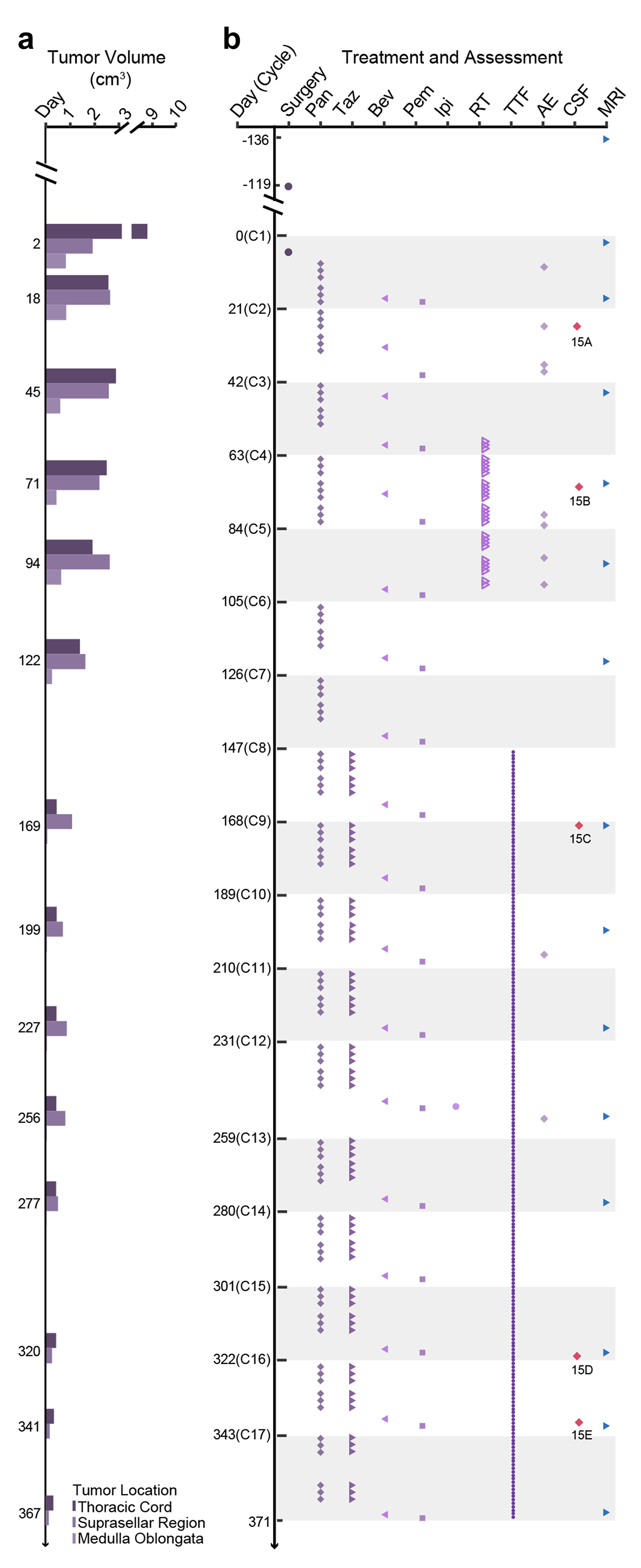

Supplement: Supplementary file 3 — Fig S2 [file 41392_2022_1274_MOESM3_ESM.tif]

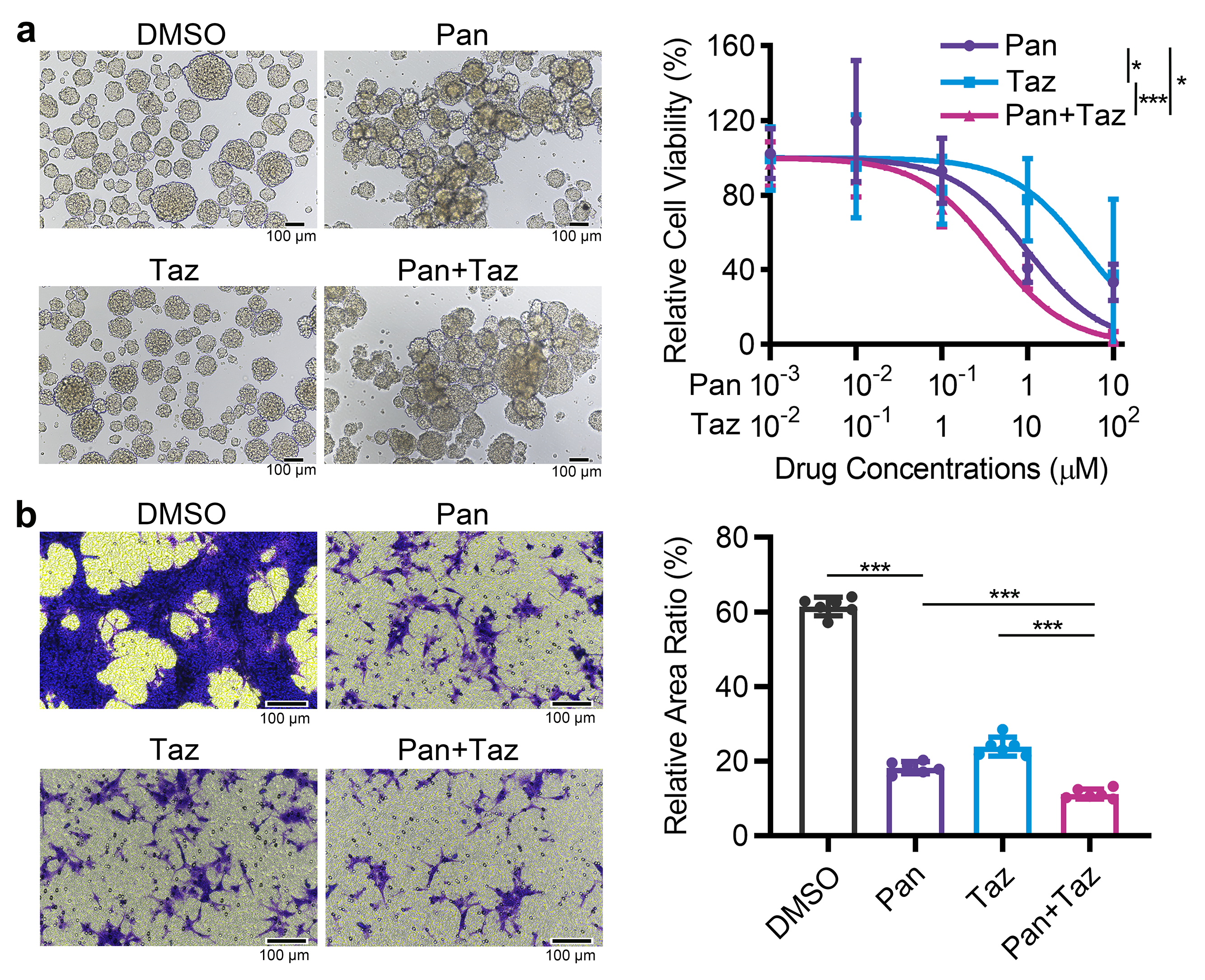

Supplement: Supplementary file 4 — Fig S3 [file 41392_2022_1274_MOESM4_ESM.tif]

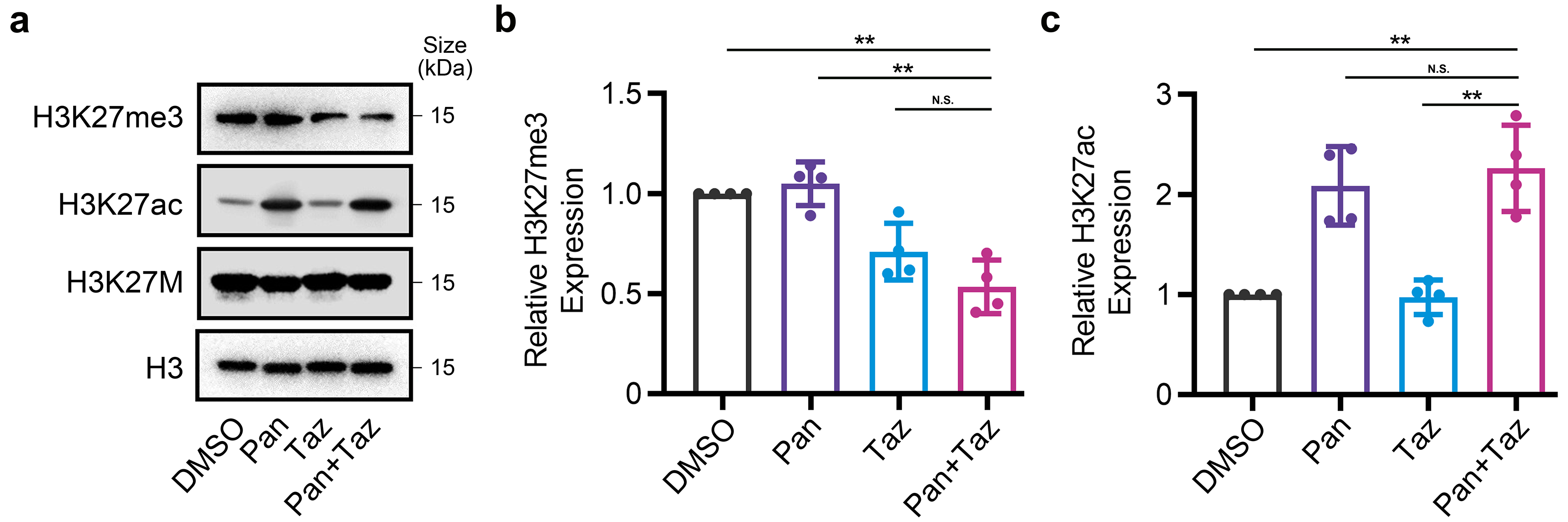

Supplement: Supplementary file 5 — Fig S4 [file 41392_2022_1274_MOESM5_ESM.tif]

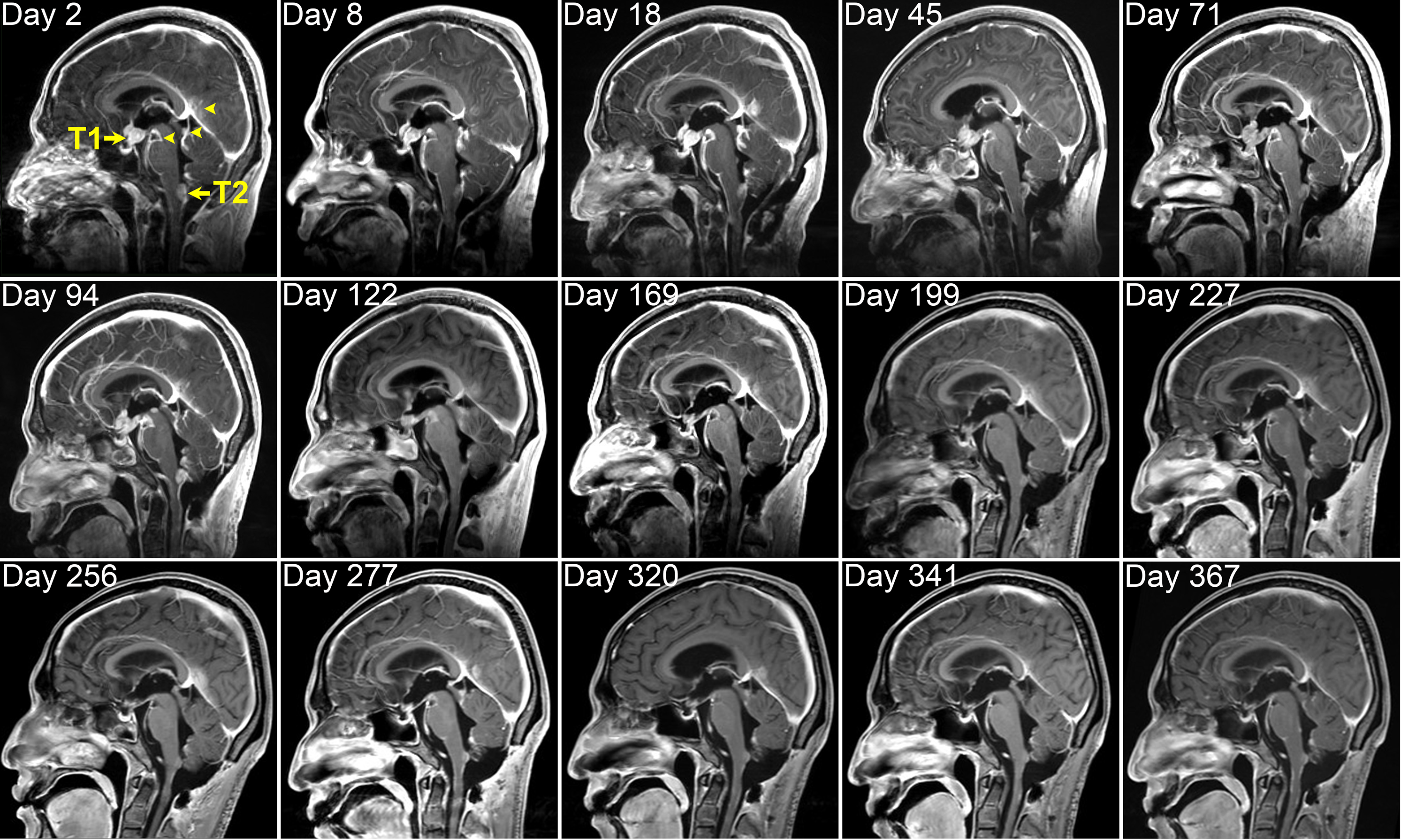

Supplement: Supplementary file 6 — Fig S5 [file 41392_2022_1274_MOESM6_ESM.tif]

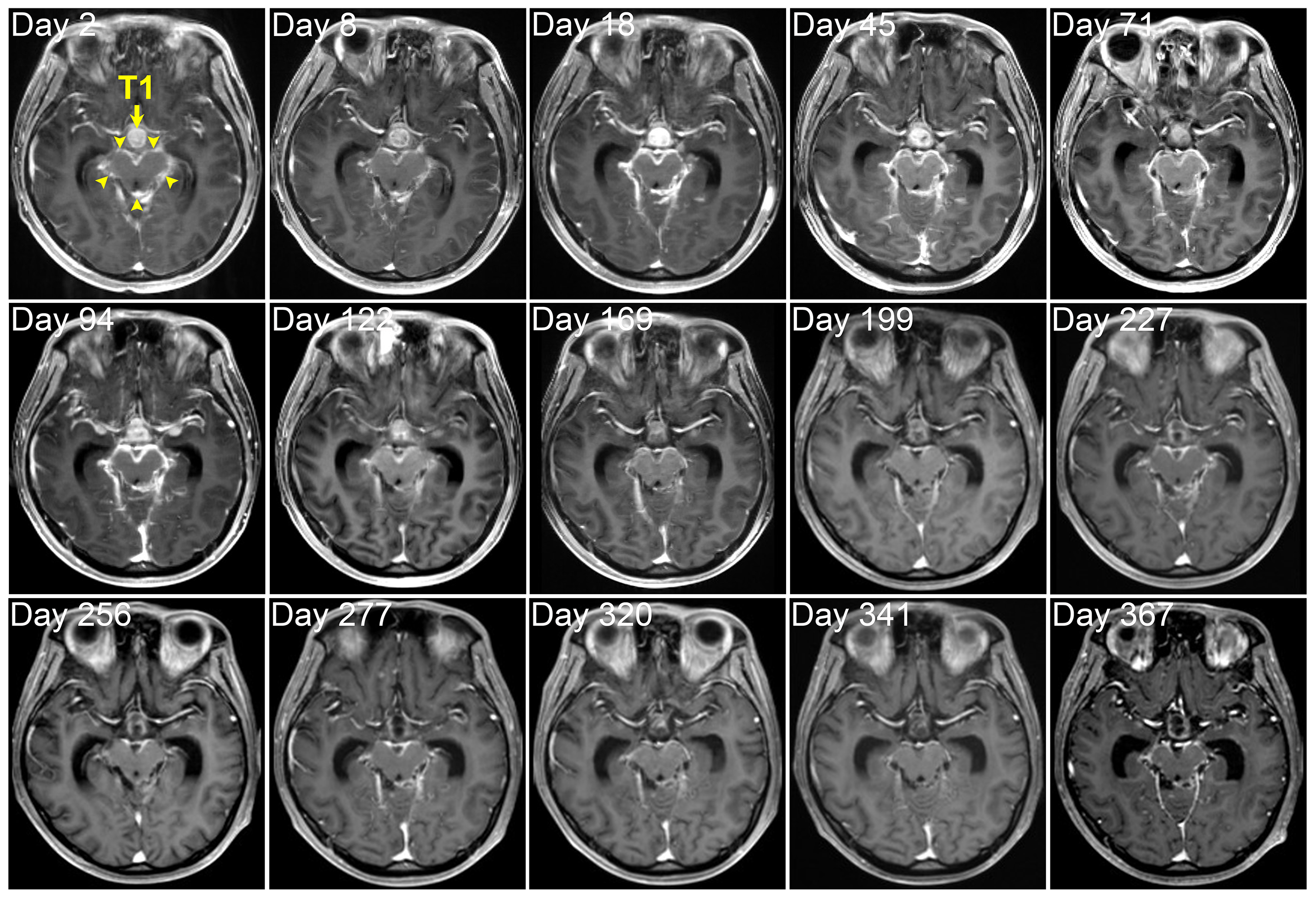

Supplement: Supplementary file 7 — Fig S6 [file 41392_2022_1274_MOESM7_ESM.tif]

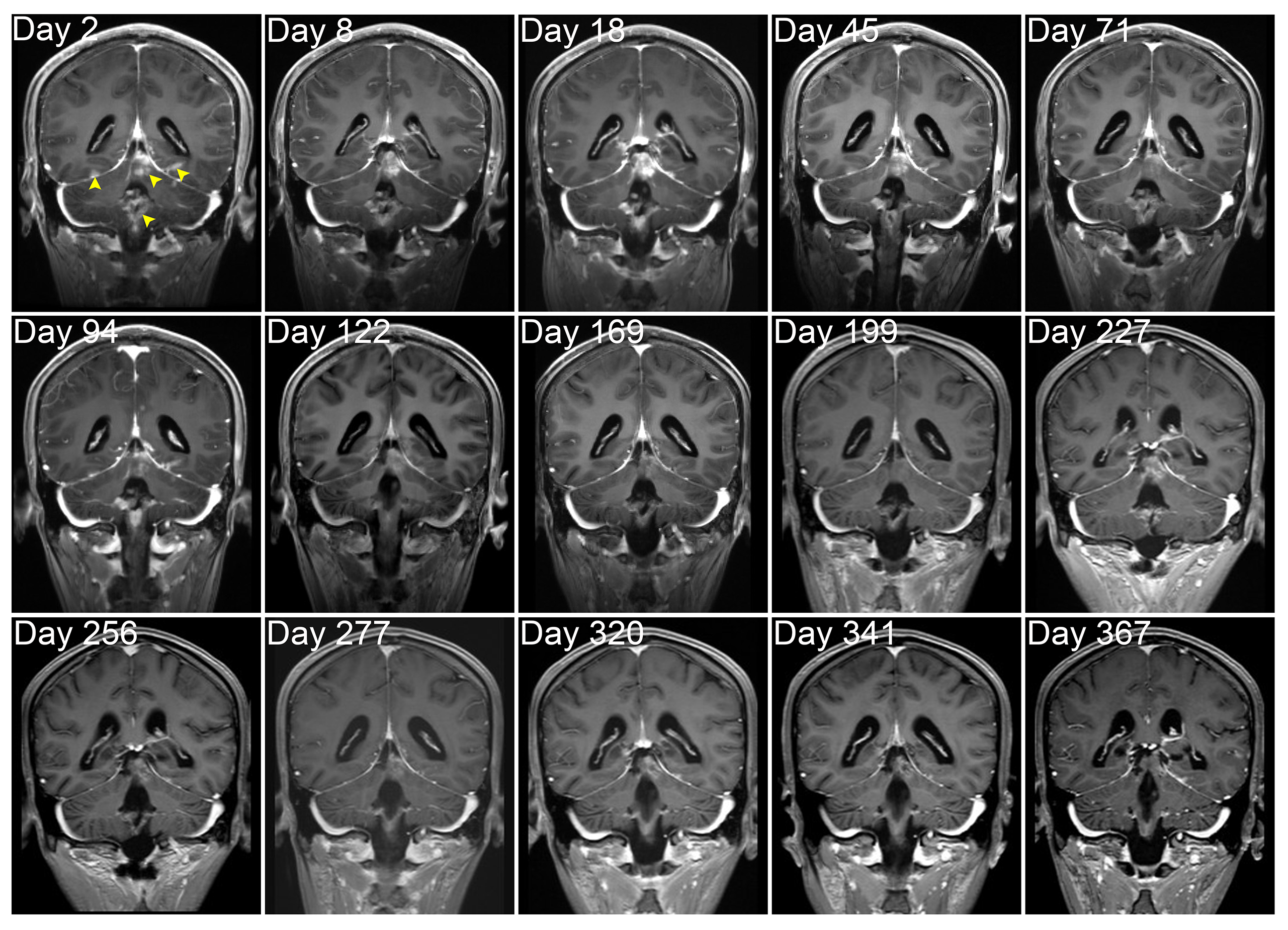

Supplement: Supplementary file 8 — Fig S7 [file 41392_2022_1274_MOESM8_ESM.tif]

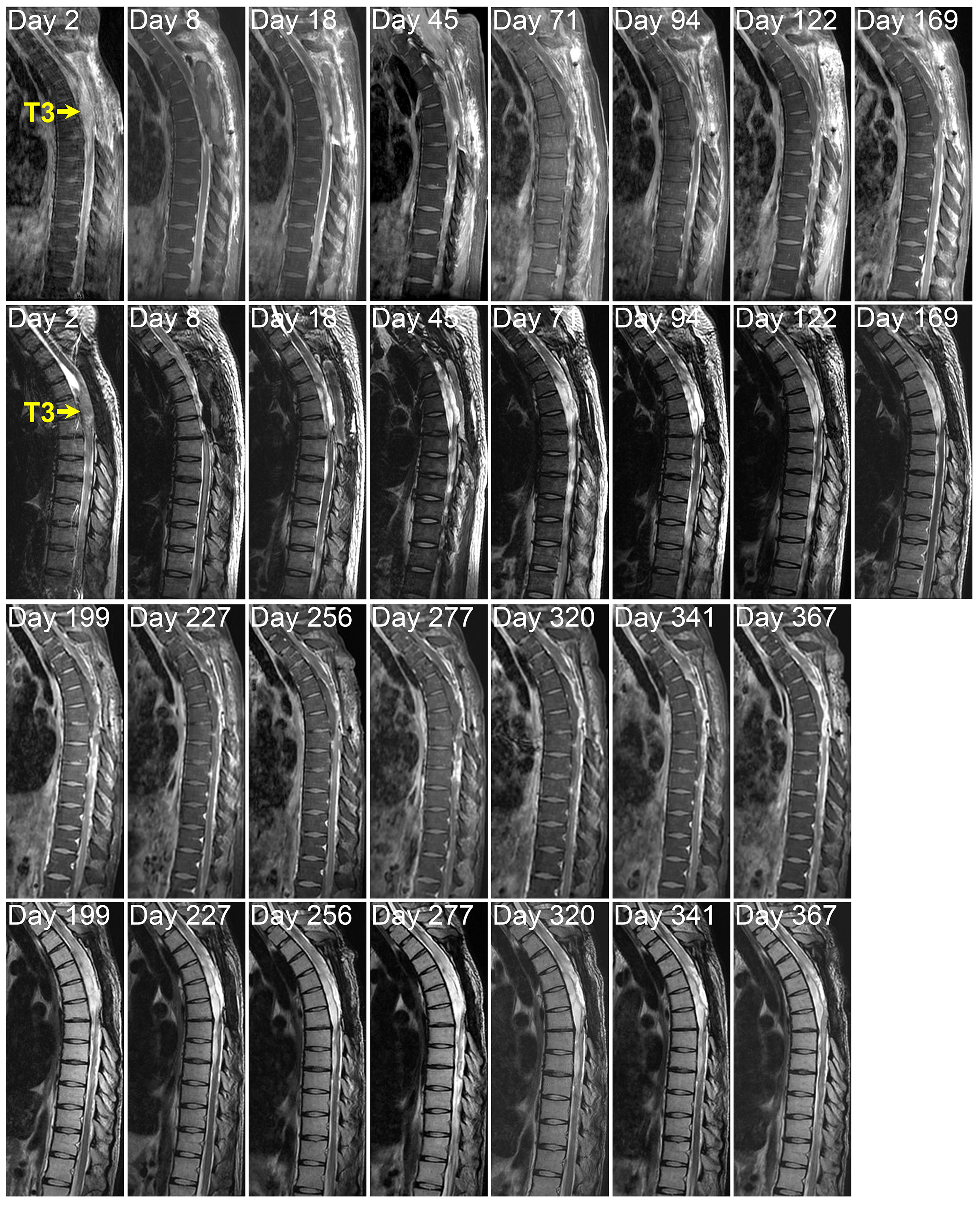

Supplement: Supplementary file 9 — Fig S8 [file 41392_2022_1274_MOESM9_ESM.tif]

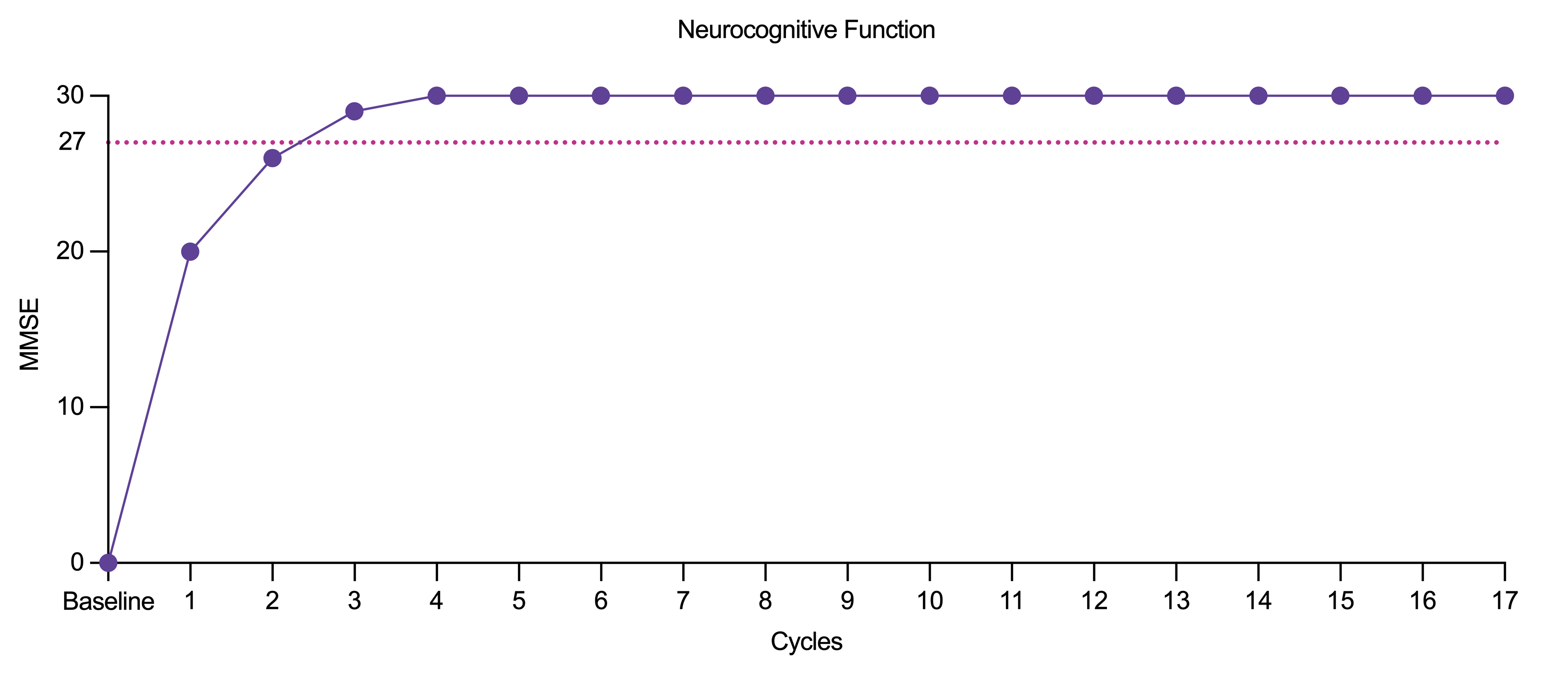

Supplement: Supplementary file 10 — Fig S9 [file 41392_2022_1274_MOESM10_ESM.tif]

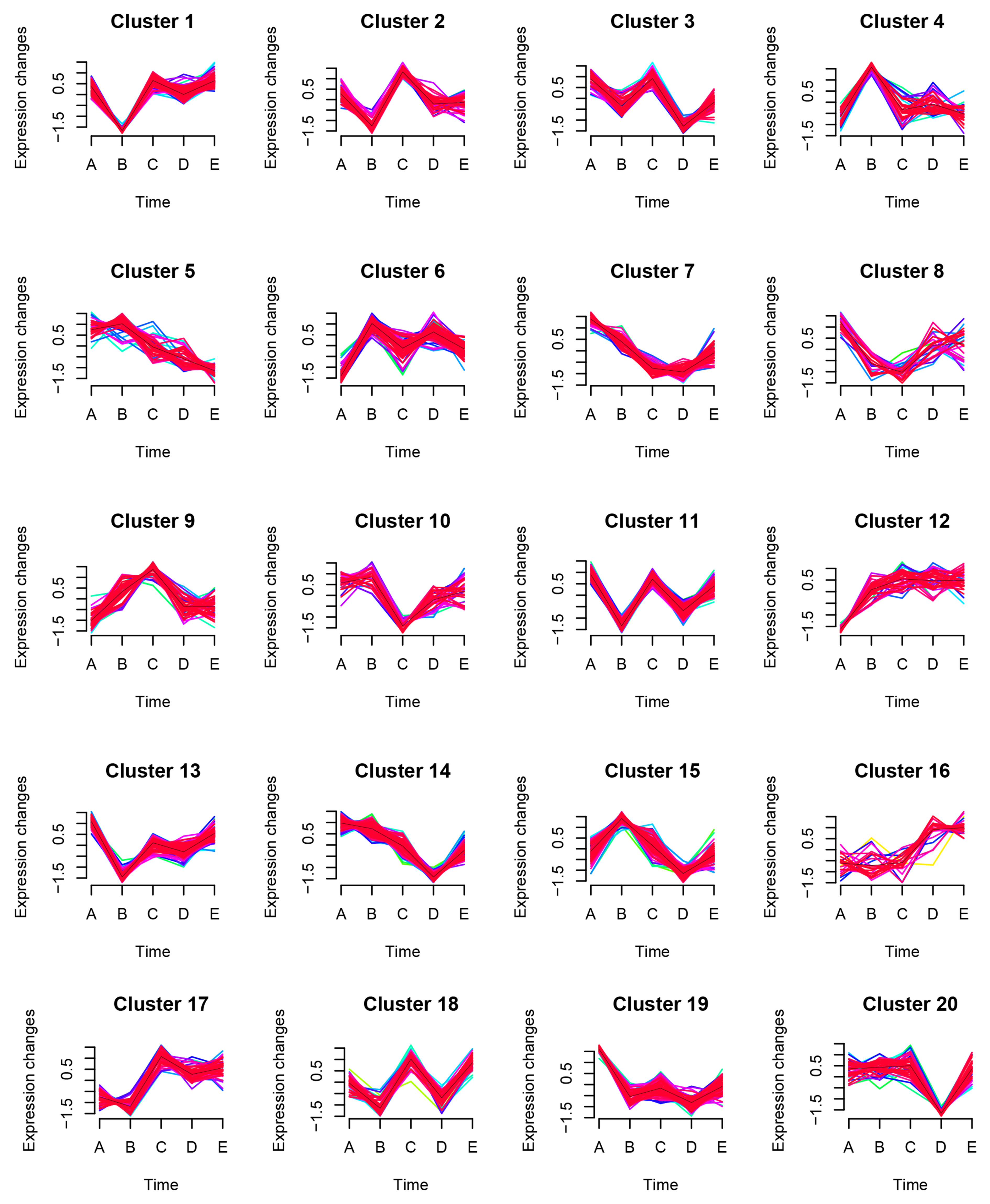

Supplement: Supplementary file 11 — Fig S10 [file 41392_2022_1274_MOESM11_ESM.tif]

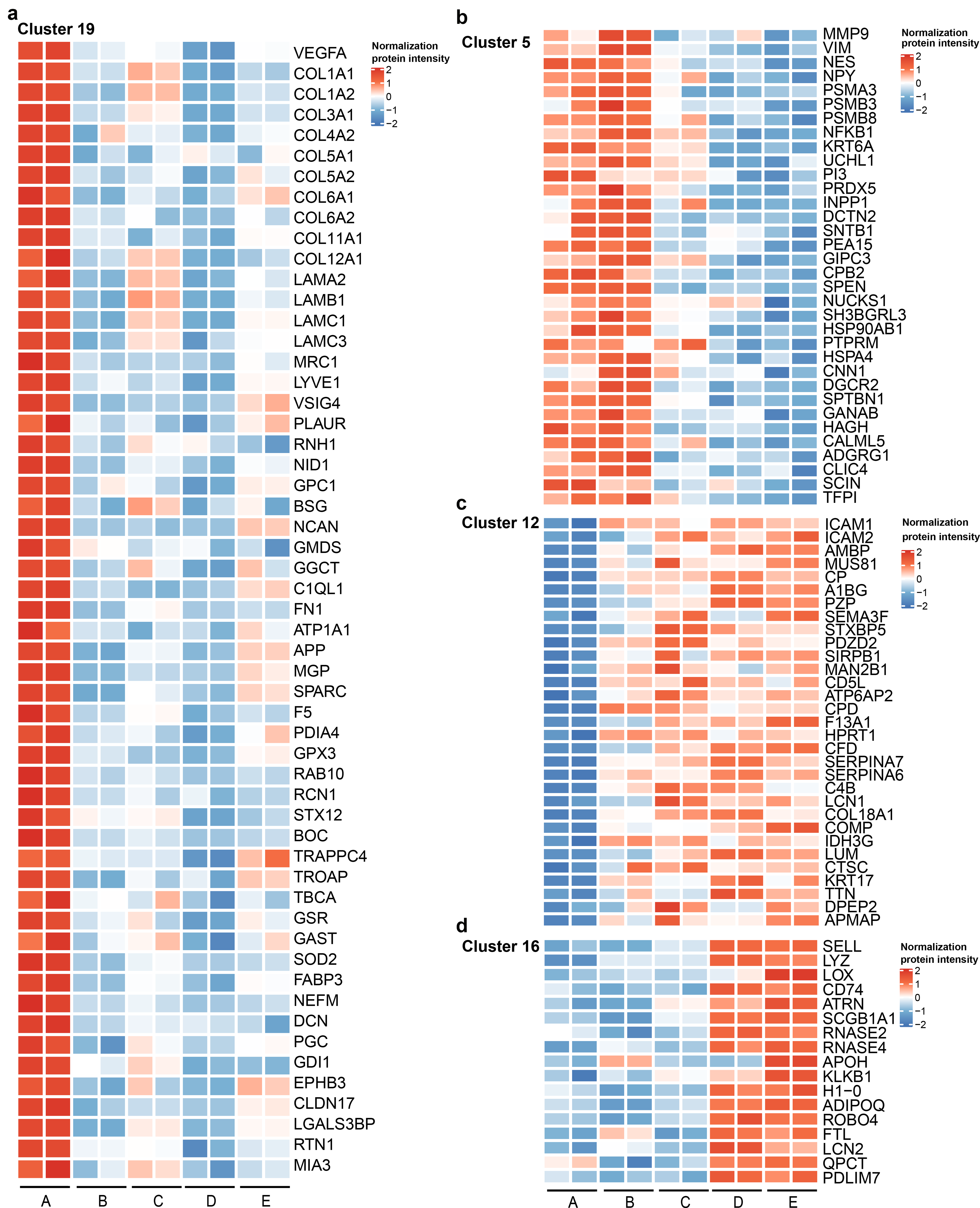

Supplement: Supplementary file 12 — Fig S11 [file 41392_2022_1274_MOESM12_ESM.tif]

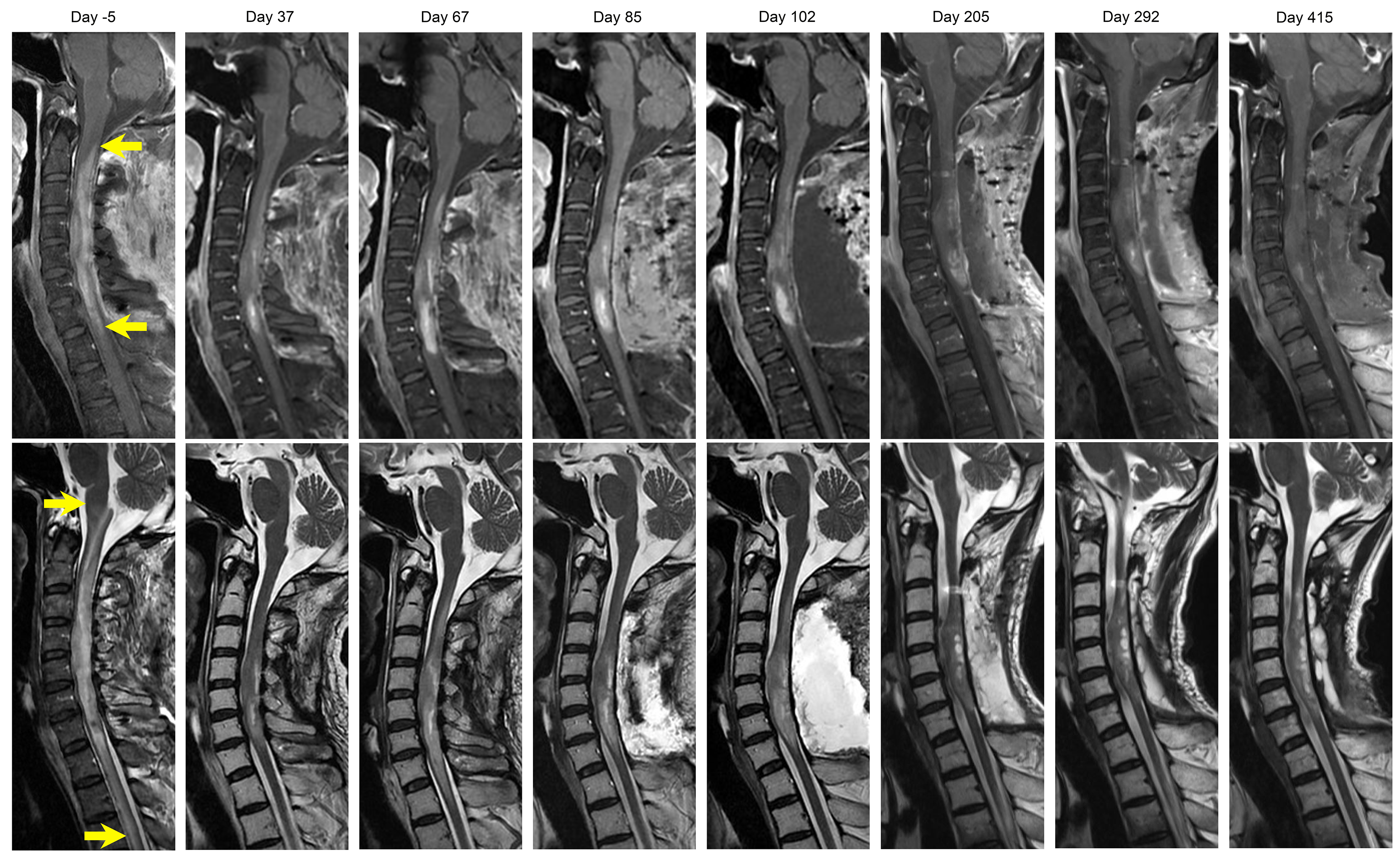

Supplement: Supplementary file 13 — Fig S12 [file 41392_2022_1274_MOESM13_ESM.tif]

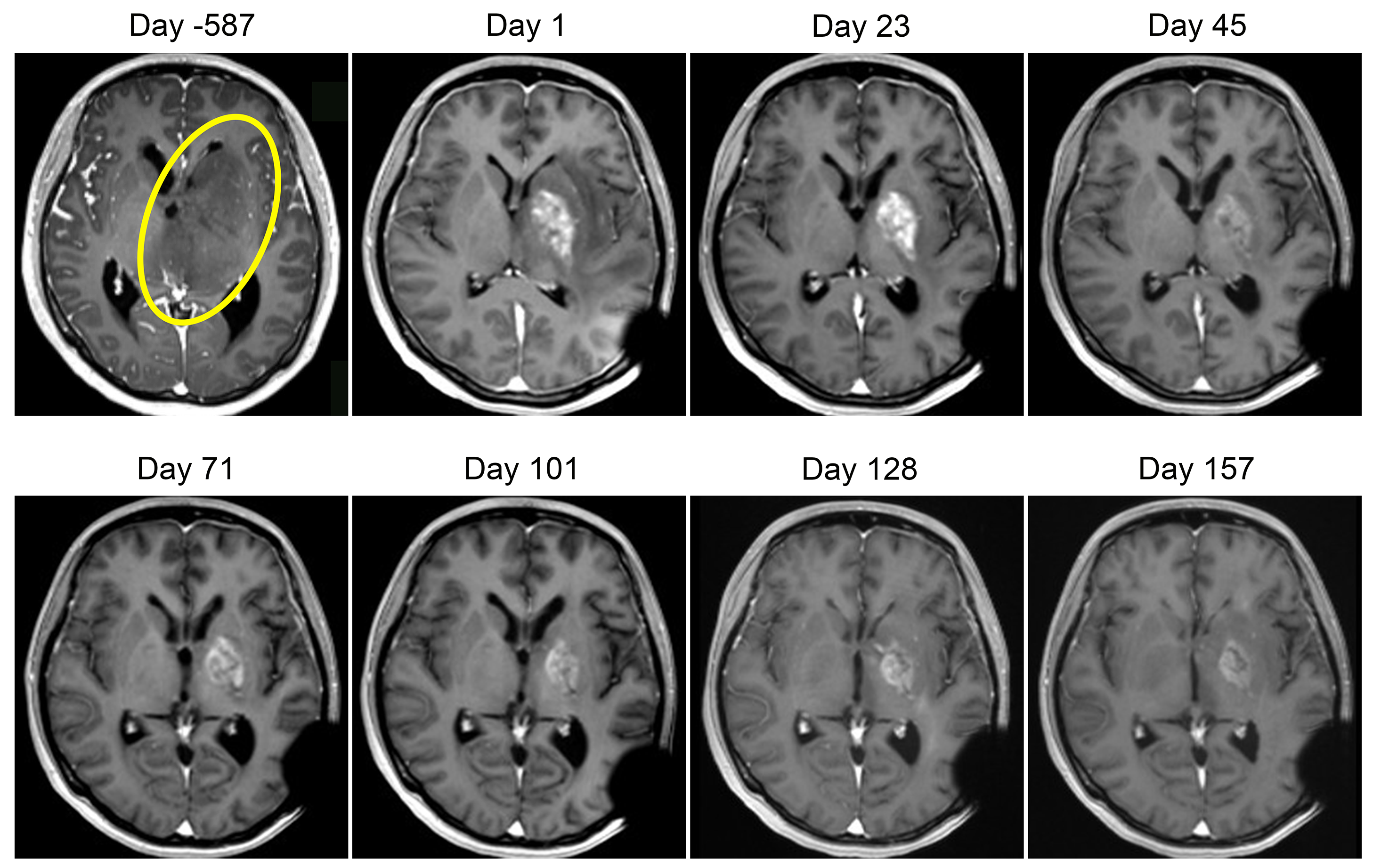

Supplement: Supplementary file 14 — Fig S13 [file 41392_2022_1274_MOESM14_ESM.tif]

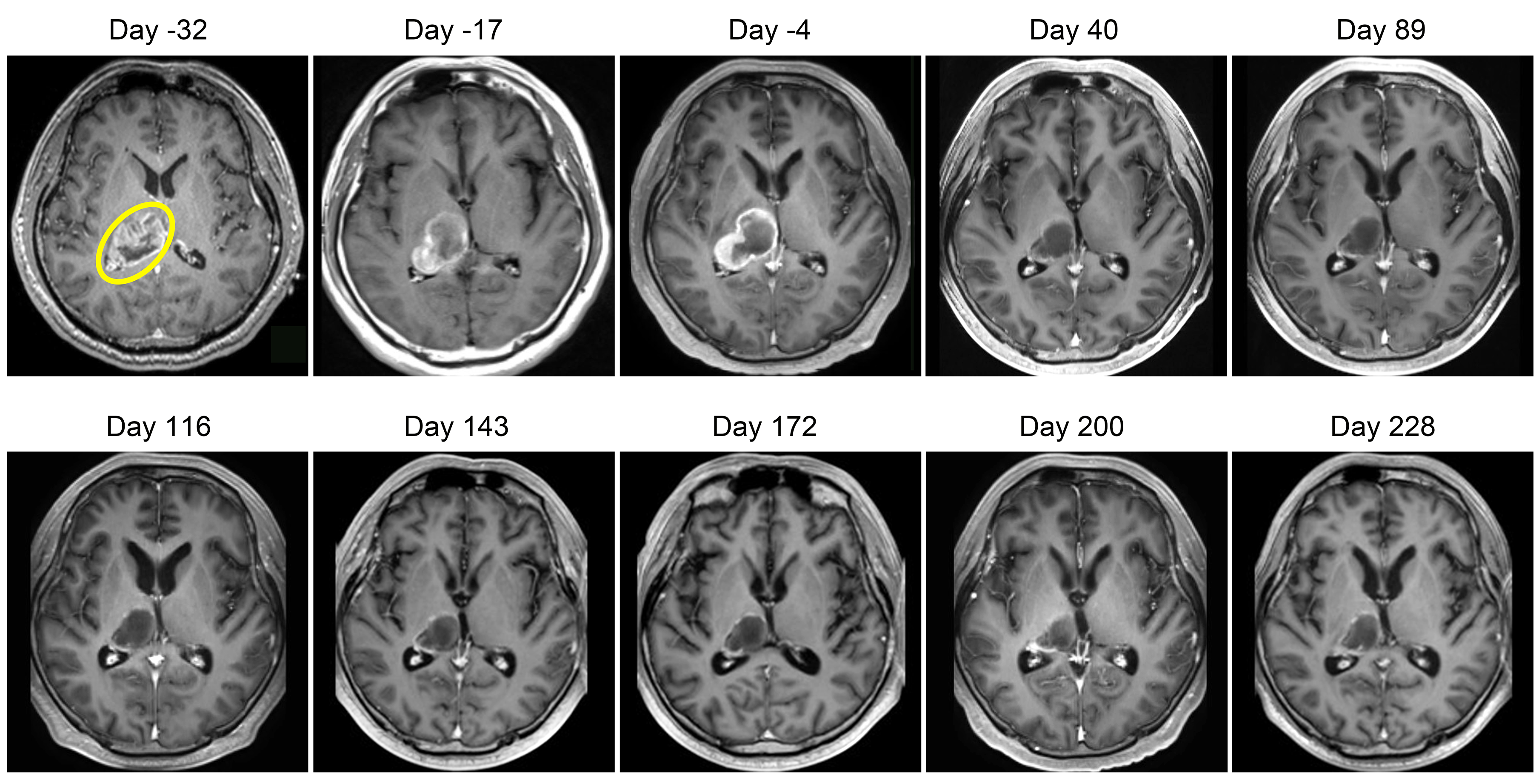

Supplement: Supplementary file 15 — Fig S14 [file 41392_2022_1274_MOESM15_ESM.tif]

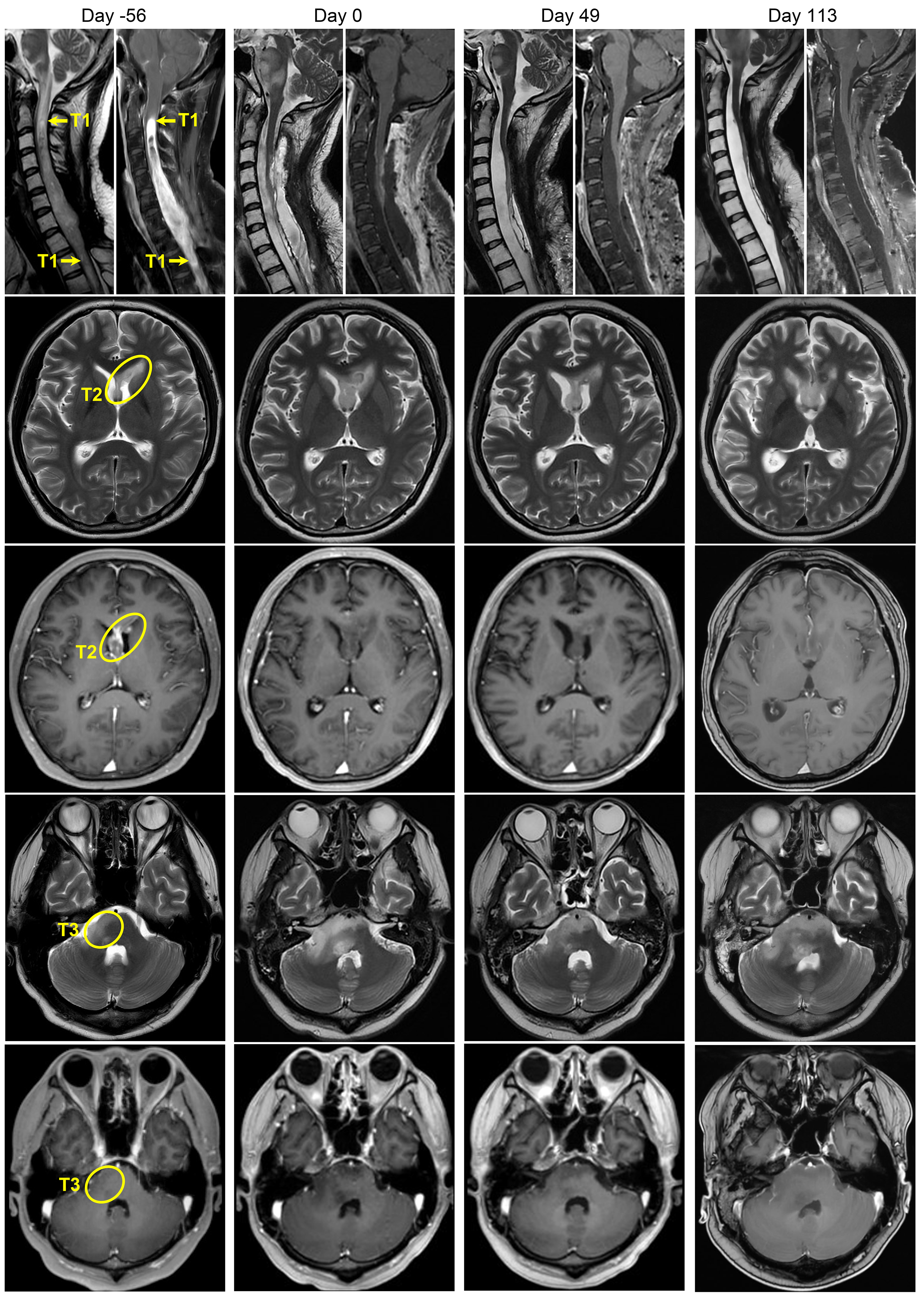

Supplement: Supplementary file 16 — Fig S15 [file 41392_2022_1274_MOESM16_ESM.tif]
