# Supplementary material for: Diffuse midline glioma treated with epigenetic agent-based immunotherapy
Source: Signal Transduct Target Ther. 2023 Jan 20;8:23. doi: 10.1038/s41392-022-01274-7 (PMC9852273; doi:10.1038/s41392-022-01274-7)
Supplement: Supplementary file 1 — Supplementary Materials [file 41392_2022_1274_MOESM1_ESM.docx]

Supplementary Materials for

Diffuse Midline Glioma Treated with Epigenetic Agent Based Immunotherapy

Linkai Jing^1,^*, M.D., Ph.D., Zhihong Qian^2,^*, Qiang Gao^1,3^*, Rui Sun^4,5^, M.S., Zili Zhen^1,3^, Guihuai Wang^1^, M.D., Xuejun Yang^1^, M.D., Haitao Li^6,7^, Ph.D., Tiannan Guo^4,5^, Ph.D., and Wei Zhang^1,3,8,#^, M.D., Ph.D.

^1^Department of Neurosurgery, Beijing Tsinghua Changgung Hospital, Tsinghua University, Beijing, P.R.China

^2^Department of Basic Medical Sciences, School of Medicine, Tsinghua University, Beijing, P.R.China

^3^School of Clinical Medicine, Tsinghua University, Beijing, P.R.China

^4^Key Laboratory of Structural Biology of Zhejiang Province, School of Life Sciences, Westlake University, Zhejiang, P.R.China

^5^Institute of Basic Medical Sciences, Westlake Institute for Advanced Study, Zhejiang, P.R.China

^6^Ministry of Education Key Laboratory of Protein Sciences, Beijing Advanced Innovation Center for Structural Biology, Beijing Frontier Research Center for Biological Structure, Department of Basic Medical Sciences, School of Medicine, Tsinghua University, Beijing, P.R.China

^7^Tsinghua-Peking Center for Life Sciences, Tsinghua University, Beijing, P.R.China

^8^IDG/McGovern Institute for Brain Research, School of Life Sciences, Tsinghua University, Beijing, P.R.China

*Dr. Linkai Jing, Zhihong Qian, and Qiang Gao contributed equally to this article.

Correspondence to: Dr. Wei Zhang, [wzhang007@tsinghua.edu.cn](mailto:wzhang007@tsinghua.edu.cn)

**This PDF file includes:**

Materials and Methods

Figures S1 to S15

Tables S1 to S6

References

Materials and Methods

Therapeutic Details

A single-patient protocol was approved by the Ethics Committee of the Beijing Tsinghua Changgung Hospital for a compassionate purpose. Written informed consent for participation was given by the patient and his family. No commercial sponsor was involved. Briefly, panobinostat was administered orally 20 mg 3 times per week (Days 1, 3, 5, 8, 10, 12) in week 1 and 2 of a 3-week cycle,**^1^** in combination with intravenously bevacizumab at 10 mg/kg and pembrolizumab at 200 mg once in week 3 of each cycle. Based on the critical condition of the patient at presentation, bevacizumab was given every two weeks through cycles 1–4. Adjuvant radiotherapy of whole brain (41.4 Gy in 23 fractions) and tumor bed boost in sellar region (10 Gy in 5 fractions) was delivered during cycles 3–5. Due to grade 3 leukopenia, neutropenia, and weight loss, panobinostat was suspended in cycle 5 (Supplementary Fig. 2b and Table 2). Since DMG cells exhibited resistance to chronic panobinostat exposure, effective combination therapies were undoubtedly necessary.**^2^** Our *in* *vitro* cell viability assay and matrigel assay indicated that THG15 cells (derived from the thoracic tumor of patient 1; See assay details below) were highly sensitive to panobinostat combined tazemetostat treatment, compared with either agent alone (Supplementary Fig. 3 and 4). Treatment with oral 400 mg tazemetostat and tumor treating fields concurrent with panobinostat were initiated from cycle 8. The dose of panobinostat was reduced to 10 mg in cycle 11 due to satisfactory therapeutic responses as well as increased alkaline phosphatase in cycle 10 (Fig. 1a and Supplementary Fig. 2, Day 199; Supplementary Table 2 and 3), but reverted to 20 mg since cycle 12 for LM progression (Supplementary Fig. 2, Day 227; Supplementary Table 3). Ipilimumab (50 mg intravenously) was introduced only once in cycle 12 for following considerations: 1. Enhancer of zeste homolog 2 inhibitor (EZH2i) could improve the efficacy of anti-cytotoxic T lymphocyte antigen 4 (CTLA-4) inhibitor and overcome ICB resistance;^3,4^ 2. Periventricular LM had slightly progressed in cycle 11; 3. All lesions sufficiently regressed afterwards (Supplementary Fig. 2 and Table 3); 4. Combination of programmed cell death protein 1 (PD-1) and CTLA-4 inhibitor has been reported with increased immune-related adverse events which may result in therapy suspension or discontinuation.**^5^** Due to grade 2 fatigue, cycle 13 was delayed by one week during which no medication was administered, and the dose of tazemetostat was reduced to 200 mg since cycle 13. The three-week treatment cycle was extended to a four-week cycle**^6^** in cycle 17. Our data cutoff date was day 371 (April 12^th^, 2022).

Safety and Adverse Event (AE) Profile

Ten AE, graded 1–3, were documented that were partially attributable to therapy (Supplementary Table 2). Grade 3 AE included anemia, leukopenia, neutropenia, and weight loss, all of which resolved following blood transfusion or panobinostat suspension. AE of lower grades included diarrhea, fatigue, thrombocytopenia and elevated liver enzymes. No AE was graded 4 or warranted treatment withdrawal.

Pathologic Examination

Formalin-fixed, paraffin-embedded tissue sections were examined with hematoxylin and eosin (HE) and immunohistochemistry staining as previously described.^7^ Primary antibodies against Ki-67 (mouse monoclonal, clone MIB-1; Labvision, Fremont, CA, USA), H3K27M (rabbit polyclonal, SAB4500352; Sigma-Aldrich, St Louis, MO, USA), H3K27me3 (rabbit polyclonal, ZA0327; ZSBIO, Beijing, China), and isocitrate dehydrogenase 1 (IDH1) R132H mutant (mouse monoclonal, clone H09, 1:500; Dianova, Hamburg, Germany) and secondary antibodies (ZSBIO) were used for immunohistochemistry staining. Immunoreaction was detected with a Leica Bond automated staining processor.

Patient-Derived Cell Cultures

Patient-derived cell cultures were generated with informed consent in compliance with the Ethics Committee of the Beijing Tsinghua Changgung Hospital. Surgical specimen derived from the thoracic tumor of patient 1 was named as THG15 (Tsinghua Glioma 15). Procedure was followed as previously described.^8,9^ Briefly, mechanically minced tissues were processed with 0.1% trypsin (Hyclone) and 10 U/mL DNaseI (Roche) at 37°C for 45 min. The tissues were triturated and passed through a 100-μm cell strainer after lysis of red blood cells and several washes. Strained cells were plated in EF medium composed of Neurobasal medium (Invitrogen), 3 mM L-glutamine (Mediatech), 1×B27 supplement (Thermo Fisher Scientific), 0.5×N2 supplement (Thermo Fisher Scientific), 2 μg/mL heparin (Sigma-Aldrich), 20 ng/mL recombinant human EGF (Sigma-Aldrich), 20 ng/mL recombinant human FGF2 (Sigma-Aldrich), and 0.5×penicillin G/streptomycin sulfate/amphotericin B complex (Mediatech). Cell cultures were fed every third day through replenishment with fresh EF medium. Cell passaging was performed following dissociation of neurospheres using NeuroCult Chemical Dissociation kit (StemCell Technologies, Vancouver, BC, CAN).

Cell Viability Assay

Panobinostat (LBH589) and Tazemetostat (EPZ-6438) were purchased from Selleck Chemicals (Houston, TX, USA) and dissolved in 0.1% DMSO before use. About 5,000 THG15 cells were seeded into each well of a 96-well plate and cultured in EF medium for 24 hours prior to drug exposure. Cells were then incubated for 72 hours with panobinostat, tazemetostat, or both at indicated concentrations in triplicates. Cell viability was determined with a cell counting kit-8 (GK10001, GLPBIO) following manufacturer’s recommendations, and the optical density was measured at 450 nm using a SPARK reader (TECAN). To determine the inhibitory response of each regimen, viability of drug-exposed cells was compared with that of cells incubated in growth media alone. Dose-response curves with a standard Hill slope of -1.0 were generated and corresponding half maximal inhibitory concentrations (IC_50_) calculated on Prism 9 (GraphPad) based on two independently performed viability assays.

Transwell Migration Assay

About 5x10^5^ THG15 cells suspended in 100 μL serum-free Dulbecco's Modified Eagle Medium (DMEM; Gibco) were seeded into each transwell insert (the upper chamber) in a 24-well plate (3422, Corning). DMEM supplemented with 20% FBS (Gibco) was added to the lower chamber, together with 1 μM panobinostat, 50 μM tazemetostat, or both in triplicates. After 48 hours of incubation, the cells were fixed in 4% paraformaldehyde (Solarbio) and stained with 1% crystal violet (Solarbio). The upper surface of the transwell floor was gently wiped with a cotton swab to remove cells that failed to migrate across the membrane. Residual cells were visualized under an inverted fluorescence microscope (Ts2R-FL, Nikon). Relative area of stained cells was quantified on ImageJ as average from three random views for each replicate.

Western Blotting Assay

To analyze epigenetic alterations in drug-exposed tumor cells, 5×10^5^ THG15 cells were incubated for 48 hours with 1 μM panobinostat, 50 μM tazemetostat, or both in 6-well plates in triplicates. Cell density post drug exposure was inspected in a bright field with an inverted fluorescence microscope (Ts2R-FL, Nikon), and representative images were captured on NIS-Elements (Nikon) with auto-exposure and auto-white balance. Cells were lysed in RIPA buffer (R0020; Solarbio, Beijing, China) per manufacturer’s protocol and prepared with 5×SDS-PAGE Sample Loading Buffer (Beyotime, Shanghai, China) 4-to-1-diluted in PBS (Solarbio). Total protein levels of cell lysates were semi-quantified on SurePAGE™ gels (Genscript, Nanjing, China) using eStain™ L1 Protein Staining system (Genscript). For western blotting, cell lysates of volumes adjusted for total protein levels were electrophoresed on a 20% Bis-Tris gel (Genscript). Separated proteins were transferred to an Immobilon^®^-P PVDF Membrane (Millipore) using an eBlot^®^ L1 Fast Wet Transfer System (Genscript). After blockade with 5% skimmed milk dissolved in TBST composed of 1×TBS (Lablead) and 0.1% TWEEN 20 (VWR), protein blots were incubated with primary antibodies against H3K27Me3 (mouse monoclonal, Ab6002, 1:1,000; Abcam, Cambridge, UK), H3K27Ac (rabbit polyclonal, Ab4729, 1:1,000; Abcam), H3K27M (rabbit monoclonal, Ab190631, 1:1,000; Abcam), or H3 (rabbit polyclonal, Ab1791, 1:5,000; Abcam), followed by horseradish peroxidase-conjugated goat anti-rabbit (BE0101, 1:10,000; EASYBIO, Beijing, China) or anti-mouse (BE0102, 1:10,000; EASYBIO) secondary antibodies. Probed blots were detected using SuperSignal^TM^ West Pico PLUS Chemiluminescent Substrate (Thermo Fisher Scientific) and imaged on ImageQuant LAS 4000 (GE). The relative intensities of blots were quantified with densitometric analysis on ImageJ from the National Institutes of Health.

Magnetic Resonance Imaging and Tumor Volume Measurement

MRI of the brain and spinal cord was performed on 3.0 Tesla scanners, with 3D T1-weighted, T2-weighted, and post-gadolinium T1-weighted sequences. Enhancing tumors were manually outlined by two independent neuroradiologists on the post-contrast T1-weighted MRI images, and the tumor volumes were calculated with ITK-SNAP as previously described.^10^

Health Related Quality of Life Assessment

Quality of life of the patient was assessed according to two quality of life questionnaires (QLQ) constructed by the European Organization for Research and Treatment of Cancer. The core QLQ-C30 consists of five functional scales, one global health and quality-of-life scale, three multi-item symptom scales, five single-item symptom measures, and a single measure of perceived financial impact of disease and treatment.^11^ The brain cancer specific QLQ-BN20 comprises four multi-trait scales and seven single-item symptom measures.^12^ Raw scale and measure scores were linearly transformed onto a 0-to-100 scale for ease of interpretation, wherein a higher score corresponded to a higher level of functioning or symptoms.^11^ All scales and measures, except for physical functioning, were scored based on the average state of health and quality of life during the *week* prior to data collection.

Proteomic Analysis of CSF

Following standard collection of CSF samples, proteins of high abundance were first depleted using High Select™ Top14 Abundant Protein Depletion Resin (Thermo Fisher Scientific) as previously described.^13^ Cleaned peptides were separated with Thermo Scientific UltiMate^TM^ 3000 RSLCnano System and analyzed with an Orbitrap Exploris 480 mass spectrometry using FAIMS-PulseDIA.^14^ The optimal compensation voltage (CV) was set to -48 V and -68V with a cycle time of 1 s per FAIMS experiment. MS1 resolution was set at 60,000 with a normalized AGC target of 300%. Mass range was set to 390–1010 Th. One full scan was followed by 12 windows with a resolution of 30,000 and a normalized AGC target of 200%. For part 1 method, the mass range was set to 520–630 Th at -68V and 630–750 Th at -48V. For part 2 method, the mass range was set to 400–520 Th at -68V and 750–1000 Th at -48V. Normalized HCD collision energy was 32%. The background was a human FASTA file downloaded from the UniProt proteome dataset on 26th January 2020. The database searching applied a library-free strategy using DIA-NN (version 1.7.8) as previously described.^14^ Raw files have been deposited to the iProX: IPX0005197001.

Statistical Analysis

One-way analysis of variance (ANOVA) was used to determine the statistical differences among protein levels at different time points, and the p-values were adjusted by the Benjamini & Hochberg method. Differentially expressed proteins through the therapy course (ANOVA adjusted *p<0.05*) were grouped into 20 clusters based on expression profiles with the use of Mfuzz package.^15^

Supplementary Fig. 1 Pathologic Diagnosis. The representative staining derived from pathologic diagnosis of the recurrent thoracic tumor (TT, upper panel) and leptomeningeal metastasis in dorsal medulla (LM, lower panel). Neovascularization as well as foci of hemorrhage and necrosis are present around tumor cells on HE staining. Both lesions had a high Ki-67 score, with tumor cells staining positive for H3K27M and negative for H3K27me3 and IDH1. TT, thoracic tumor; LM, leptomeningeal metastasis; HE, hematoxylin and eosin; H3K27me3, trimethylated H3K27; IDH1, isocitrate dehydrogenase 1.

**Supplementary Fig. 2** **Response of Tumors to Epigenetic Therapy.** **a.** shows volumes of the three lesions, thoracic cord, suprasellar region, and medulla oblongata, on each MRI assessment that were measured by ITK-SNAP.^10^ **b.** shows the timeline for treatment, which consisted of epigenetic agents (panobinostat and tazemetostat), VEGFA inhibitor bevacizumab, ICB (pembrolizumab and ipilimumab), radiotherapy, and tumor treating fields. First occurrences of treatment-related AE (refer to Supplementary Table 2 for details), analyses of CSF, and MRI assessments are also dated on the timeline. Timeline for the current treatment spanned from January 11, 2021 (Day 1), four months after the initial diagnosis, to data cutoff on January 16, 2022 (Day 371), comprising 17 cycles of 21 or 28 days. CSF samples were denoted 15A–E in a chronological order for ease of reference (see Fig. 1b). Pan, panobinostat; Taz, tazemetostat; Bev, bevacizumab; Pem, pembrolizumab; Ipi, ipilimumab; RT, radiotherapy; TTF, tumor treating fields; AE, adverse events; CSF, cerebrospinal fluid; MRI, magnetic resonance imaging; LM, leptomeningeal metastasis; VEGFA, vascular endothelial growth factor A; ICB, immune checkpoint blockade.

**Supplementary Fig. 3** **Response of Patient-Derived Tumor Cells to Epigenetic Therapy.** Panel **a** shows dose-response curves generated with measurements of cell viability after 72 hours of exposure to panobinostat, tazemetostat, or both at indicated concentrations. Viability results from two independently performed assays are shown as pooled means±standard deviations (n=6). The drug solvent, 0.1% DMSO, was found not to affect cell viability under relevant conditions (data not shown). The IC_50_ value for each regimen was compared with each other with the extra sum-of-squares F test: *, *p<0.05*; ***, *p<0.001*. Representative images demonstrating tumor cell viability under microscopic examination after 48 hours of incubation with 1 μM panobinostat or 50 μM tazemetostat, that approximated the respective estimated IC_50_, or 1 μM panobinostat+50 μM tazemetostat are shown on the left of Panel **a**. Panel **b** shows effect of panobinostat and tazemetostat on cell migration in transwells. Tumor cells that had migrated across the transwell membrane after 48 hours of incubation in 1 μM panobinostat, 50 μM tazemetostat, or both were stained and visualized under the microscope as shown on the left of Panel **b**. Area of stained cells relative to field was quantified on ImageJ as average from three random views for each replicate. Results are shown as means±standard deviations (n=6). Unpaired t-tests were performed: ***, *p<0.001*. DMSO, dimethyl sulfoxide; Pan, panobinostat; Taz, tazemetostat; IC_50_, half maximal inhibitory concentration.

**Supplementary Fig. 4** **Molecular Response of Patient-Derived Tumor Cells to Epigenetic Therapy.** Panel **a** shows expression of H3K27me3 and H3K27ac, assessed by western blot assay, in patient-derived tumor cells after 48 hours of incubation in 1 μM panobinostat, 50 μM tazemetostat, or both, and DMSO control. Levels of H3K27me3 and H3K27ac relative to DMSO control are shown in Panels **b** and **c**, respectively. Student’s t test revealed significantly decreased expression of H3K27me3 and increased H3K27ac in tumor cells treated with combined panobinostat and tazemetostat (n=4; N.S., not significant; **, *p<0.01*). Levels of H3 and H3K27M in tumor cells were also measured with western blot assay (Panel **a**). DMSO, dimethyl sulfoxide; Pan, panobinostat; Taz, tazemetostat; H3K27me3, trimethylated H3K27; H3K27ac, acetylated H3K27.

Supplementary Fig. 5 Response of Intracranial LM on Imaging (Sagittal View). Sagittal view of contrast-enhanced T1-weighted MRI images on the days indicated in the upper left of each image. Multiple foci of linear leptomeningeal enhancement are highlighted with arrowheads. T1 and T2 represent suprasellar LM and medulla oblongata nodules in baseline MRI (Day 2). Refer to Supplementary Fig. 6 and 7 for axial and coronal views. Note that some of the images (on Day 2, 18, 94, 122, 227, and 367) are also shown in Fig. 1a.

Supplementary Fig. 6 Response of Intracranial LM on Imaging (Axial View). Axial view of contrast-enhanced T1-weighted MRI images on the days indicated in the upper left of each image. Multiple foci of linear leptomeningeal and periventricular enhancement are highlighted with arrowheads and T1 represents suprasellar LM in baseline MRI (Day 2). Refer to Supplementary Fig. 5 and 7 for sagittal and coronal views. Note that some of the images (on Day 2, 18, 94, 122, 227, and 367) are also shown in Fig. 1a.

Supplementary Fig. 7 Response of Intracranial LM on Imaging (Coronal View). Coronal view of contrast-enhanced T1-weighted MRI images on the days indicated in the upper left of each image. Multiple foci of linear leptomeningeal and periventricular enhancement are highlighted with arrowheads in baseline MRI (Day 2). Refer to Supplementary Fig. 5 and 6 for sagittal and axial views. Note that some of the images (on Day 2, 18, 94, 122, 227, and 367) are also shown in Fig. 1a.

Supplementary Fig. 8 Response of Thoracic Tumor on Imaging (Sagittal View). Contrast-enhanced T1-weighted and T2-weighted MRI images on the days indicated in the upper left of each image. T3 represents recurrent thoracic tumor in baseline MRI (Day 2). Refer to Supplementary Table 3 for measurement of tumor volume at each assessment. Note that some of the images (on Day 2, 18, 94, 122, 227, and 367) are also shown in Fig. 1a.

Supplementary Fig. 9 Neurocognitive Function. Neurocognitive function of the patient was assessed according to the Mini–Mental State Examination (MMSE) with scores ranging from 0 to 30. A score of, or above, 27 points indicates a normal cognition.

Supplementary Fig. 10 Clustered Trends of Differential Protein Expression in CSF. Following depletion of proteins of high abundance, CSF proteins with differential expression through the treatment course (ANOVA adjusted *p<0.05*) were grouped into 20 clusters based on patterns of relative expression with the use of Mfuzz package. Trends in relative expression of proteins in all 20 clusters are shown.

Supplementary Fig. 11 Full Lists of Differentially Expressed Proteins in CSF. Panels a–d show heatmaps illustrating the relative expression of proteins in CSF samples collected at various time points during treatment (refer to Supplementary Fig. 2b for the timeline). Proteins differentially expressed through the therapy course (ANOVA adjusted *p<0.05*) were grouped into 20 clusters based on expression profiles (refer to Supplementary Fig. 9 for cluster characteristics) with the use of Mfuzz package. Note that results from proteins of interest are also shown in Fig. 1b.

Supplementary Fig. 12 Clinical Course of Patient 2. The patient was a 53-year-old male presented with a sudden paraplegia, paresthesia and urinary incontinence. Contrast-enhanced T1-weighted and T2-weighted MRI scans depicted the site of tumor on indicated days with details described below. Cervical DMG was diagnosed from previous tumor biopsy which was performed in another hospital. He was admitted to our hospital with radiographic and clinical signs of tumor progression (Day -5, primary tumor in T1-weighted images and edema in T2-weighted images were highlighted with arrows). He received radiotherapy (45 Gy in 25 fractions) with concurrent epigenetic agent-based immunotherapy and VEGFA inhibitor bevacizumab in a 3-week cycle. Laminectomy was performed on Day 71. Following treatment, he experienced significant improvement in motor and sensory functions of his lower extremities and recovery of bladder control. These clinical improvements were accompanied by significantly decreased tumor volume in the cervical cord (Day 415). The patient survived 16+ months from diagnosis and 15+ months from epigenetic agent-based immunotherapy with the last follow up on October 2022.

Supplementary Fig. 13 Clinical Course of Patient 3. The patient was a 25-year-old female presented with right hemiplegia and aphasia. Contrast-enhanced T1-weighted MRI scans depicted the site of tumor on indicated days with details described below. Left thalamic DMG was diagnosed from previous tumor biopsy which was performed in another hospital (Day -587, primary tumor was highlighted with the circle) followed by standard radio- and chemo- therapy. Her neurological symptoms progressively worsened. Re-radiotherapy was delivered without any clinical or radiographic improvement, and her symptoms kept deteriorating. MRI indicated left thalamic tumor progression (Day 1). She received epigenetic agent-based immunotherapy and VEGFA inhibitor bevacizumab in a 3 or 4-week cycle. Following treatment, she experienced significant improvement in right-sided motor functions and she could ambulate independently. Her language skills improved in aspects including naming and fluency and she enjoyed conversations. These clinical improvements were accompanied by remarkably regressed tumor volume in the left thalamus (Day 157). Her tumor eventually progressed due to the lack supply of epigenetic agents during COVID-19 pandemic. She died 27 months from diagnosis and 8 months after epigenetic agent-based immunotherapy.

Supplementary Fig. 14 Clinical Course of Patient 4. The patient was a 44-year-old male presented with left upper and lower limb weakness. Contrast-enhanced T1-weighted MRI scans depicted the site of tumor on indicated days with details described below. Right thalamic DMG was diagnosed from previous tumor resection which was performed in another hospital (Day -32, before surgery, primary tumor was highlighted with the circle; Day -17, after surgery). He was referred to our hospital with radiographic and clinical signs of tumor progression (Day -4). He received radiotherapy (54 Gy in 27 fractions) followed by tumor treating fields with concurrent epigenetic agents-based immunotherapy and VEGFA inhibitor bevacizumab in a 3 or 4-week cycle. Following treatment, he experienced extraordinary recovery of his left-sided motor functions. His quality of life improved dramatically and went back to normal daily activities. He was able to climb mountains and actively engage in social activities. These clinical improvements were accompanied by remarkably diminished tumor volume in the right thalamus (Day 228). However, the patient enrolled in a clinical trial of CAR T cell therapy and discontinued our epigenetic agent-based immunotherapy. He eventually died from tumor progression 16 months from diagnosis, 15 months after epigenetic agent-based immunotherapy.

Supplementary Fig. 15 Clinical Course of Patient 5. T2-weighted and contrast-enhanced T1-weighted MRI scans depicted the sites of tumors 1 through 3 (T1-T3) on indicated days with details described below. The patient was a 25-year-old man presented with progressive weakness of left upper limb and neck pain. Cervical cord DMG was diagnosed from the first tumor resection which was performed in Germany in September 2017. He received standard chemo- and radio- therapy after the first surgery. Due to tumor recurrence, the second surgery was performed in Germany in February 2019 with the same pathologic diagnosis. Without any adjuvant therapy after the second surgery, the patient enrolled in a clinical trial of ONC201 in adults with recurrent H3K27M-mutant glioma from May 2019 to March 2020 in the United States. In September 2020, he presented with paraplegia, urinary and fecal incontinence, right facial paralysis and was referred to our hospital. MRI scan indicated cervical cord tumor recurrence (T1) with multifocal brain metastases (T2 and T3) (Day -56). Subtotal resection of cervical cord tumor was performed on Day -38. However, his brain metastases significantly progressed after the third surgery with worsened neurological symptoms (Day 0). Panobinostat and bevacizumab were given in a 3-week cycle. After 2 cycles of treatment, brain metastases of T2 and T3 remarkably regressed (Day 49) with improved neurological symptoms. Unfortunately, brain metastases eventually progressed after cycle 4. MRI scan on Day 113 was the last imaging before his death on Day 163 indicating the increased tumor burden of brain metastases. He survived 43 months from diagnosis and 5 months from panobinostat combined bevacizumab treatment.

Supplementary Table 1 Summary of Clinical Parameters of Patients

| **Patient**  **No.** | **Gender** | **Age**  **(years)** | **Primary or Recurrent** | **Tumor Location** | **Histone H3** | **OS**  **(months)** |
| --- | --- | --- | --- | --- | --- | --- |
| 1 | Male | 28 | Recurrent | Thoracic Cord with LM | H3F3A mut | 20 |
| 2 | Male | 53 | Recurrent | Cervical Cord | H3F3A mut | 16+ |
| 3 | Female | 25 | Recurrent | Left Thalamus | H3F3A mut | 27 |
| 4 | Male | 44 | Recurrent | Right Thalamus | H3F3A mut | 16 |
| 5 | Male | 25 | Recurrent | Cervical Cord with Multifocal Brain Metastases | H3F3A mut | 43 |

Supplementary Table 2 Treatment Related Adverse Events

| **Day (Cycle)** | **Event** | **System Organ Class** | **Baseline Grade** | **CTCAE Grade** | **Treatment Strategy** | **Outcome** |
| --- | --- | --- | --- | --- | --- | --- |
| 9  (C1) | Diarrhea | Gastrointestinal disorders | Normal | 1 | Drug therapy | Resolved |
| 26  (C2) | Alanine aminotransferase increased | Hepatobiliary  disorders | Normal | 2 | Conservative treatment | Resolved |
| 37  (C2) | Anemia | Blood and lymphatic system disorders | Normal | 3 | Transfusion | Resolved |
| 39  (C2) | Aspartate aminotransferase increased | Hepatobiliary  disorders | Normal | 1 | Conservative treatment | Resolved |
| 80  (C4) | Platelet count decreased | Blood and lymphatic system disorders | Normal | 2 | Transfusion | Improved |
| 83  (C4) | White blood cell decreased | Blood and lymphatic system disorders | Normal | 3 | Panobinostat suspension | Resolved |
| 92  (C5) | Neutrophil count decreased | Blood and lymphatic system disorders | Normal | 3 | Panobinostat suspension | Resolved |
| 100  (C5) | Weight loss | Metabolism and nutrition disorders | 1 | 3 | Panobinostat suspension | Resolved |
| 206  (C10) | Alkaline phosphatase increased | Investigations | Normal | 2 | Conservative treatment | Resolved |
| 253  (C12) | Fatigue | General disorders and administration site conditions | 1 | 2 | Conservative treatment | Resolved |

Supplementary Table 3 Volumes of Tumors Through the Course of Therapy (mm^3^)

| **Day (Cycle)** | **Thoracic Cord**  **(T3)** | **Suprasellar Region**  **(T1)** | **Medulla Oblongata**  **(T2)** |
| --- | --- | --- | --- |
| 2 (C1) | 9057 | 1905 | 829 |
| 18 (C1) | 2538 | 2606 | 840 |
| 45 (C3) | 2842 | 2557 | 603 |
| 71 (C4) | 2476 | 2174 | 446 |
| 94 (C5) | 1896 | 2591 | 637 |
| 122 (C6) | 1394 | 1615 | 265 |
| 169 (C9) | 459 | 1078 | 77 |
| 199 (C10) | 454 | 707 | 61 |
| 227 (C11) | 446 | 864 | 66 |
| 256 (C12) | 442 | 803 | 59 |
| 277 (C13) | 435 | 508 | 43 |
| 320 (C15) | 433 | 270 | 21 |
| 341 (C16) | 346 | 177 | 18 |
| 367 (C17) | 324 | 137 | 14 |

Supplementary Table 4 Health Related Quality of Life

**QLQ-C30 Scores**

| **_QLQ Score_ ^Cycle^** | **Baseline** | **C1** | **C2** | **C3** | **C4** | **C5** | **C6** | **C7** | **C8** | **C9** | **C10** | **C11** | **C12** | **C13** | **C14** | **C15** | **C16** | **C17** |
| --- | --- | --- | --- | --- | --- | --- | --- | --- | --- | --- | --- | --- | --- | --- | --- | --- | --- | --- |
| Physical functioning | 0 | 0 | 7 | 7 | 7 | 7 | 7 | 13 | 13 | 20 | 20 | 20 | 20 | 27 | 27 | 27 | 27 | 27 |
| Role functioning | 0 | 0 | 33 | 33 | 33 | 50 | 50 | 50 | 50 | 67 | 67 | 67 | 67 | 100 | 100 | 100 | 100 | 100 |
| Cognitive functioning | 33 | 33 | 67 | 83 | 100 | 100 | 100 | 100 | 100 | 100 | 100 | 100 | 100 | 100 | 100 | 100 | 100 | 100 |
| Emotional functioning | 0 | 33 | 42 | 67 | 75 | 75 | 75 | 100 | 100 | 100 | 100 | 100 | 100 | 100 | 100 | 100 | 100 | 100 |
| Social functioning | 0 | 33 | 33 | 67 | 83 | 83 | 83 | 83 | 83 | 100 | 100 | 100 | 100 | 100 | 100 | 100 | 100 | 100 |
| Global quality of life | 0 | 25 | 25 | 25 | 25 | 33 | 33 | 33 | 42 | 67 | 67 | 67 | 67 | 67 | 67 | 75 | 83 | 100 |
| Fatigue | 100 | 100 | 67 | 67 | 67 | 67 | 67 | 33 | 33 | 33 | 33 | 33 | 67 | 33 | 0 | 0 | 0 | 0 |
| Pain | 0 | 33 | 33 | 33 | 33 | 33 | 33 | 33 | 33 | 33 | 33 | 33 | 33 | 0 | 0 | 0 | 0 | 0 |
| Nausea and Vomiting | 33 | 17 | 17 | 67 | 100 | 67 | 67 | 67 | 67 | 33 | 33 | 33 | 0 | 0 | 0 | 0 | 0 | 0 |
| Dyspnea | 100 | 67 | 67 | 33 | 33 | 33 | 33 | 0 | 0 | 0 | 0 | 0 | 0 | 0 | 0 | 0 | 0 | 0 |
| Sleep disturbance | 100 | 67 | 33 | 0 | 0 | 0 | 0 | 0 | 0 | 0 | 0 | 0 | 0 | 0 | 0 | 0 | 0 | 0 |
| Appetite loss | 100 | 67 | 67 | 100 | 100 | 100 | 100 | 100 | 100 | 67 | 67 | 67 | 67 | 67 | 67 | 67 | 33 | 0 |
| Constipation | 0 | 0 | 0 | 0 | 0 | 0 | 0 | 0 | 0 | 0 | 0 | 0 | 0 | 0 | 0 | 0 | 0 | 0 |
| Diarrhea | 0 | 33 | 0 | 0 | 0 | 0 | 0 | 0 | 0 | 0 | 0 | 0 | 0 | 0 | 0 | 0 | 0 | 0 |
| Financial impact | 100 | 67 | 33 | 33 | 0 | 0 | 0 | 0 | 0 | 0 | 0 | 0 | 0 | 0 | 0 | 0 | 0 | 0 |

**QLQ-BN20 Scores**

| **_QLQ Score_ ^Cycle^** | **Baseline** | **C1** | **C2** | **C3** | **C4** | **C5** | **C6** | **C7** | **C8** | **C9** | **C10** | **C11** | **C12** | **C13** | **C14** | **C15** | **C16** | **C17** |
| --- | --- | --- | --- | --- | --- | --- | --- | --- | --- | --- | --- | --- | --- | --- | --- | --- | --- | --- |
| Future uncertainty | 100 | 67 | 50 | 33 | 25 | 25 | 25 | 25 | 25 | 0 | 0 | 0 | 0 | 0 | 0 | 0 | 0 | 0 |
| Visual disorder | 33 | 33 | 33 | 22 | 22 | 22 | 0 | 0 | 0 | 0 | 0 | 0 | 0 | 0 | 0 | 0 | 0 | 0 |
| Motor dysfunction | 100 | 67 | 56 | 44 | 44 | 44 | 44 | 44 | 44 | 33 | 33 | 33 | 33 | 33 | 33 | 33 | 33 | 33 |
| Communication deficit | 67 | 22 | 22 | 11 | 0 | 0 | 0 | 0 | 0 | 0 | 0 | 0 | 0 | 0 | 0 | 0 | 0 | 0 |
| Headaches | 67 | 33 | 0 | 0 | 0 | 0 | 0 | 0 | 0 | 0 | 0 | 0 | 0 | 0 | 0 | 0 | 0 | 0 |
| Seizures | 0 | 0 | 0 | 0 | 0 | 0 | 0 | 0 | 0 | 0 | 0 | 0 | 0 | 0 | 0 | 0 | 0 | 0 |
| Drowsiness | 33 | 33 | 0 | 0 | 0 | 0 | 0 | 0 | 0 | 0 | 0 | 0 | 0 | 0 | 0 | 0 | 0 | 0 |
| Hair loss | 0 | 0 | 0 | 0 | 100 | 100 | 67 | 33 | 33 | 33 | 33 | 33 | 33 | 0 | 0 | 0 | 0 | 0 |
| Itchy skin | 0 | 0 | 0 | 0 | 0 | 0 | 0 | 0 | 0 | 0 | 0 | 0 | 0 | 0 | 0 | 0 | 0 | 0 |
| Weakness of legs | 67 | 100 | 100 | 100 | 100 | 100 | 100 | 100 | 100 | 100 | 100 | 100 | 100 | 100 | 100 | 100 | 100 | 100 |
| Bladder control | 67 | 100 | 100 | 100 | 100 | 100 | 100 | 100 | 100 | 100 | 100 | 100 | 100 | 100 | 100 | 100 | 100 | 100 |

Supplementary Table 5 Reference Articles of Key Differentially Expressed Proteins

| **Protein** | **Protein Name** | **References** |
| --- | --- | --- |
| VEGFA | Vascular endothelial growth factor A | ^16^, ^17^, ^18^ |
| COL1A1 | Collagen alpha-1(I) chain | ^19^, ^20^, ^21^, ^22^ |
| COL1A2 | Collagen alpha-2(I) chain | ^23^ |
| COL3A1 | Collagen alpha-1(III) chain | ^24^ |
| COL4A2 | Collagen alpha-2(IV) chain | ^25^ |
| COL5A1 | Collagen alpha-1(V) chain | ^26^ |
| COL5A2 | Collagen alpha-2(V) chain | ^27^ |
| COL6A1 | Collagen alpha-1(VI) chain | ^28^ |
| COL6A2 | Collagen alpha-2(VI) chain | ^27^ |
| COL11A1 | Collagen alpha-1(XI) chain | ^29^ |
| COL12A1 | Collagen alpha-1(XII) chain | ^30^ |
| LAMA2 | Laminin subunit alpha-2 | ^31^, ^32^ |
| LAMB1 | Laminin subunit beta-1 | ^33^ |
| LAMC1 | Laminin subunit gamma-1 | ^34^ |
| LAMC3 | Laminin subunit gamma-3 | ^35^ |
| MMP9 | Matrix metalloproteinase-9 | ^36^, ^37^ |
| VIM | Vimentin | ^38^, ^39^, ^40^ |
| NES | Nestin | ^41^, ^42^ |
| PZP | Pregnancy zone protein | ^43^, ^44^ |
| PSMA3 | Proteasome subunit alpha type-3 | ^45^, ^46^ |
| PSMB3 | Proteasome subunit beta type-3 | ^47^ |
| PSMB8 | Proteasome subunit beta type-8 | ^48^ |
| MRC1 | Macrophage mannose receptor 1 | ^49^, ^50^, ^51^ |
| VSIG4 | V-set and immunoglobulin domain-containing protein 4 | ^52^, ^53^ |
| PLAUR | Urokinase plasminogen activator surface receptor | ^54^, ^55^, ^56^ |
| ICAM1 | Intercellular adhesion molecule 1 | ^57^, ^58^, ^59^ |
| ICAM2 | Intercellular adhesion molecule 2 | ^60^, ^61^ |
| SELL | L-selectin | ^62^, ^63^ |
| LYZ | Lysozyme C | ^64^, ^65^ |
| ATRN | Attractin | ^66^, ^67^ |

Supplementary Table 6 Additional Review or Research Articles of Significant Pertinence

| **Title** | **Agents Involved** | **Reference** | |
| --- | --- | --- | --- |
| Epigenomic reprogramming as a driver of malignant glioma | / | | ^68^ |
| Pervasive H3K27 acetylation leads to ERV expression and a therapeutic vulnerability in H3K27M gliomas | Panobinostat | | ^69^ |
| Therapeutic strategies for diffuse midline glioma from high-throughput combination drug screening | Panobinostat, marizomib | | ^70^ |
| Phase II study of panobinostat in combination with bevacizumab for recurrent glioblastoma and anaplastic glioma | Panobinostat, bevacizumab | | ^6^ |
| Functionally defined therapeutic targets in diffuse intrinsic pontine glioma | Panobinostat | | ^2^ |
| EZH2 is a potential therapeutic target for H3K27M-mutant pediatric gliomas | EZH2i | | ^71^ |
| Simultaneous disruption of PRC2 and enhancer function underlies histone H3.3-K27M oncogenic activity in human hindbrain neural stem cells | Tazemetostat | | ^72^ |
| Modulation of EZH2 expression in T cells improves efficacy of anti-CTLA-4 therapy | EZH2i, ipilimumab | | ^4^ |
| An evolutionarily conserved function of polycomb silences the MHC Class I antigen presentation pathway and enables immune evasion in cancer | EZH2i | | ^3^ |
| The emerging role of epigenetic therapeutics in immuno-oncology | Various epigenetic agents and ICB | | ^73^ |
| Effect of nivolumab vs bevacizumab in patients with recurrent glioblastoma | Nivolumab, bevacizumab | | ^74^ |
| Mechanisms and therapeutic implications of hypermutation in gliomas | Temozolomide, pembrolizumab | | ^75^ |

References

1	Garnock-Jones, K. P. Panobinostat: first global approval. *Drugs* **75**, 695-704 (2015).

2	Grasso, C. S. *et al.* Functionally defined therapeutic targets in diffuse intrinsic pontine glioma. *Nat Med* **21**, 555-559 (2015).

3	Burr, M. L. *et al.* An Evolutionarily Conserved Function of Polycomb Silences the MHC Class I Antigen Presentation Pathway and Enables Immune Evasion in Cancer. *Cancer Cell* **36**, 385-401.e388 (2019).

4	Goswami, S. *et al.* Modulation of EZH2 expression in T cells improves efficacy of anti-CTLA-4 therapy. *J Clin Invest* **128**, 3813-3818 (2018).

5	Carlino, M. S., Larkin, J. & Long, G. V. Immune checkpoint inhibitors in melanoma. *Lancet* **398**, 1002-1014 (2021).

6	Lee, E. Q. *et al.* Phase II study of panobinostat in combination with bevacizumab for recurrent glioblastoma and anaplastic glioma. *Neuro Oncol* **17**, 862-867 (2015).

7	Yao, J., Wang, L., Ge, H., Yin, H. & Piao, Y. Diffuse midline glioma with H3 K27M mutation of the spinal cord: A series of 33 cases. *Neuropathology* **41**, 183-190 (2021).

8	Zhang, W. *et al.* Combination of oncolytic herpes simplex viruses armed with angiostatin and IL-12 enhances antitumor efficacy in human glioblastoma models. *Neoplasia* **15**, 591-599 (2013).

9	Chen, W. J. *et al.* The different role of YKL-40 in glioblastoma is a function of MGMT promoter methylation status. *Cell Death Dis* **11**, 668 (2020).

10	Yushkevich, P. A. *et al.* User-guided 3D active contour segmentation of anatomical structures: significantly improved efficiency and reliability. *Neuroimage* **31**, 1116-1128 (2006).

11	Aaronson, N. K. *et al.* The European Organization for Research and Treatment of Cancer QLQ-C30: a quality-of-life instrument for use in international clinical trials in oncology. *J Natl Cancer Inst* **85**, 365-376 (1993).

12	Osoba, D. *et al.* The development and psychometric validation of a brain cancer quality-of-life questionnaire for use in combination with general cancer-specific questionnaires. *Qual Life Res* **5**, 139-150 (1996).

13	Echan, L. A., Tang, H. Y., Ali-Khan, N., Lee, K. & Speicher, D. W. Depletion of multiple high-abundance proteins improves protein profiling capacities of human serum and plasma. *Proteomics* **5**, 3292-3303 (2005).

14	Cai, X. *et al.* PulseDIA: Data-Independent Acquisition Mass Spectrometry Using Multi-Injection Pulsed Gas-Phase Fractionation. *J Proteome Res* **20**, 279-288 (2021).

15	Kumar, L. & M, E. F. Mfuzz: a software package for soft clustering of microarray data. *Bioinformation* **2**, 5-7 (2007).

16	Chinot, O. L., Wick, W. & Cloughesy, T. Bevacizumab for newly diagnosed glioblastoma. *N Engl J Med* **370**, 2049 (2014).

17	Gilbert, M. R., Sulman, E. P. & Mehta, M. P. Bevacizumab for newly diagnosed glioblastoma. *N Engl J Med* **370**, 2048-2049 (2014).

18	Manneh Kopp, R. A. *et al.* Correlation of radiological and immunochemical parameters with clinical outcome in patients with recurrent glioblastoma treated with Bevacizumab. *Clin Transl Oncol* **21**, 1413-1423 (2019).

19	Mammoto, T. *et al.* Role of collagen matrix in tumor angiogenesis and glioblastoma multiforme progression. *Am J Pathol* **183**, 1293-1305 (2013).

20	Elia, I. *et al.* Breast cancer cells rely on environmental pyruvate to shape the metastatic niche. *Nature* **568**, 117-121 (2019).

21	Kaur, A. *et al.* Remodeling of the Collagen Matrix in Aging Skin Promotes Melanoma Metastasis and Affects Immune Cell Motility. *Cancer Discov* **9**, 64-81 (2019).

22	Keerthivasan, S. *et al.* Homeostatic functions of monocytes and interstitial lung macrophages are regulated via collagen domain-binding receptor LAIR1. *Immunity* **54**, 1511-1526 (2021).

23	Sun, S. *et al.* Identification of COL1A1 as an invasion‑related gene in malignant astrocytoma. *Int J Oncol* **53**, 2542-2554 (2018).

24	Gao, Y. F. *et al.* COL3A1 and SNAP91: novel glioblastoma markers with diagnostic and prognostic value. *Oncotarget* **7**, 70494-70503 (2016).

25	Lukashova-v Zangen, I. *et al.* Ependymoma gene expression profiles associated with histological subtype, proliferation, and patient survival. *Acta Neuropathol* **113**, 325-337 (2007).

26	Gu, S. *et al.* COL5A1 Serves as a Biomarker of Tumor Progression and Poor Prognosis and May Be a Potential Therapeutic Target in Gliomas. *Front Oncol* **11**, 752694 (2021).

27	Zhang, L., Wang, L., Yang, H., Li, C. & Fang, C. Identification of potential genes related to breast cancer brain metastasis in breast cancer patients. *Biosci Rep* **41** (2021).

28	Lin, H. *et al.* Identification of COL6A1 as the Key Gene Associated with Antivascular Endothelial Growth Factor Therapy in Glioblastoma Multiforme. *Genet Test Mol Biomarkers* **25**, 334-345 (2021).

29	Vázquez-Villa, F. *et al.* COL11A1/(pro)collagen 11A1 expression is a remarkable biomarker of human invasive carcinoma-associated stromal cells and carcinoma progression. *Tumour Biol* **36**, 2213-2222 (2015).

30	Jiang, X. *et al.* COL12A1, a novel potential prognostic factor and therapeutic target in gastric cancer. *Mol Med Rep* **20**, 3103-3112 (2019).

31	Albrengues, J. *et al.* Neutrophil extracellular traps produced during inflammation awaken dormant cancer cells in mice. *Science* **361** (2018).

32	Zhang, M. *et al.* Immune-related genes LAMA2 and IL1R1 correlate with tumor sites and predict poor survival in pancreatic adenocarcinoma. *Future Oncol* **17**, 3061-3076 (2021).

33	Chen, Q. *et al.* MiR-124-5p inhibits the growth of high-grade gliomas through posttranscriptional regulation of LAMB1. *Neuro Oncol* **16**, 637-651 (2014).

34	Liu, J., Liu, D., Yang, Z. & Yang, Z. High LAMC1 expression in glioma is associated with poor prognosis. *Onco Targets Ther* **12**, 4253-4260 (2019).

35	Lesseur, C. *et al.* Genome-wide association analyses identify new susceptibility loci for oral cavity and pharyngeal cancer. *Nat Genet* **48**, 1544-1550 (2016).

36	Tabouret, E. *et al.* MMP2 and MMP9 as candidate biomarkers to monitor bevacizumab therapy in high-grade glioma. *Neuro Oncol* **17**, 1174-1176 (2015).

37	Hormigo, A. *et al.* YKL-40 and matrix metalloproteinase-9 as potential serum biomarkers for patients with high-grade gliomas. *Clin Cancer Res* **12**, 5698-5704 (2006).

38	Kalluri, R. & Weinberg, R. A. The basics of epithelial-mesenchymal transition. *J Clin Invest* **119**, 1420-1428 (2009).

39	Richardson, A. M. *et al.* Vimentin Is Required for Lung Adenocarcinoma Metastasis via Heterotypic Tumor Cell-Cancer-Associated Fibroblast Interactions during Collective Invasion. *Clin Cancer Res* **24**, 420-432 (2018).

40	Tian, H. *et al.* AKT-induced lncRNA VAL promotes EMT-independent metastasis through diminishing Trim16-dependent Vimentin degradation. *Nat Commun* **11**, 5127, doi:10.1038/s41467-020-18929-0 (2020).

41	Bagó, J. R. *et al.* Tumor-homing cytotoxic human induced neural stem cells for cancer therapy. *Sci Transl Med* **9** (2017).

42	Kleeberger, W. *et al.* Roles for the stem cell associated intermediate filament Nestin in prostate cancer migration and metastasis. *Cancer Res* **67**, 9199-9206 (2007).

43	Jensen, P. E. & Stigbrand, T. Differences in the proteinase inhibition mechanism of human alpha 2-macroglobulin and pregnancy zone protein. *Eur J Biochem* **210**, 1071-1077 (1992).

44	Kumar, R. *et al.* Genetic ablation of pregnancy zone protein promotes breast cancer progression by activating TGF-β/SMAD signaling. *Breast Cancer Res Treat* **185**, 317-330 (2021).

45	Maupin-Furlow, J. A. & Ferry, J. G. A proteasome from the methanogenic archaeon Methanosarcina thermophila. *J Biol Chem* **270**, 28617-28622 (1995).

46	Liu, J. *et al.* Immuno-oncological role of 20S proteasome alpha-subunit 3 in aggravating the progression of esophageal squamous cell carcinoma. *Eur J Immunol* **52**, 338-351 (2022).

47	Jacquemont, C. & Taniguchi, T. Proteasome function is required for DNA damage response and fanconi anemia pathway activation. *Cancer Res* **67**, 7395-7405 (2007).

48	Arima, K. *et al.* Proteasome assembly defect due to a proteasome subunit beta type 8 (PSMB8) mutation causes the autoinflammatory disorder, Nakajo-Nishimura syndrome. *Proc Natl Acad Sci U S A* **108**, 14914-14919 (2011).

49	Wu, Y. *et al.* Spatiotemporal Immune Landscape of Colorectal Cancer Liver Metastasis at Single-Cell Level. *Cancer Discov* **12**, 134-153 (2022).

50	Tanaka, S. *et al.* CD206 Expression in Induced Microglia-Like Cells From Peripheral Blood as a Surrogate Biomarker for the Specific Immune Microenvironment of Neurosurgical Diseases Including Glioma. *Front Immunol* **12**, 670131 (2021).

51	Zeiner, P. S. *et al.* Distribution and prognostic impact of microglia/macrophage subpopulations in gliomas. *Brain Pathol* **29**, 513-529 (2019).

52	Vogt, L. *et al.* VSIG4, a B7 family-related protein, is a negative regulator of T cell activation. *J Clin Invest* **116**, 2817-2826 (2006).

53	Li, J. *et al.* VSIG4 inhibits proinflammatory macrophage activation by reprogramming mitochondrial pyruvate metabolism. *Nat Commun* **8**, 1322 (2017).

54	Zeng, F. *et al.* Plasminogen Activator Urokinase Receptor Implies Immunosuppressive Features and Acts as an Unfavorable Prognostic Biomarker in Glioma. *Oncologist* **26**, e1460-e1469 (2021).

55	Ji, H. *et al.* Novel Immune-Related Gene-Based Signature Characterizing an Inflamed Microenvironment Predicts Prognosis and Radiotherapy Efficacy in Glioblastoma. *Front Genet* **12**, 736187 (2021).

56	Li, J., Fan, H., Zhou, X., Xiang, Y. & Liu, Y. Prognostic Significance and Gene Co-Expression Network of PLAU and PLAUR in Gliomas. *Front Oncol* **11**, 602321 (2021).

57	Boyd, A. W., Wawryk, S. O., Burns, G. F. & Fecondo, J. V. Intercellular adhesion molecule 1 (ICAM-1) has a central role in cell-cell contact-mediated immune mechanisms. *Proc Natl Acad Sci U S A* **85**, 3095-3099 (1988).

58	Lyck, R. *et al.* T-cell interaction with ICAM-1/ICAM-2 double-deficient brain endothelium in vitro: the cytoplasmic tail of endothelial ICAM-1 is necessary for transendothelial migration of T cells. *Blood* **102**, 3675-3683 (2003).

59	Zumwalde, N. A., Domae, E., Mescher, M. F. & Shimizu, Y. ICAM-1-dependent homotypic aggregates regulate CD8 T cell effector function and differentiation during T cell activation. *J Immunol* **191**, 3681-3693 (2013).

60	Steiner, O. *et al.* Differential roles for endothelial ICAM-1, ICAM-2, and VCAM-1 in shear-resistant T cell arrest, polarization, and directed crawling on blood-brain barrier endothelium. *J Immunol* **185**, 4846-4855 (2010).

61	Reiss, Y., Hoch, G., Deutsch, U. & Engelhardt, B. T cell interaction with ICAM-1-deficient endothelium in vitro: essential role for ICAM-1 and ICAM-2 in transendothelial migration of T cells. *Eur J Immunol* **28**, 3086-3099 (1998).

62	Watson, H. A. *et al.* L-Selectin Enhanced T Cells Improve the Efficacy of Cancer Immunotherapy. *Front Immunol* **10**, 1321 (2019).

63	Mohammed, R. N. *et al.* L-selectin Is Essential for Delivery of Activated CD8(+) T Cells to Virus-Infected Organs for Protective Immunity. *Cell Rep* **14**, 760-771 (2016).

64	Ragland, S. A. & Criss, A. K. From bacterial killing to immune modulation: Recent insights into the functions of lysozyme. *PLoS Pathog* **13**, e1006512 (2017).

65	Ferraboschi, P., Ciceri, S. & Grisenti, P. Applications of Lysozyme, an Innate Immune Defense Factor, as an Alternative Antibiotic. *Antibiotics (Basel)* **10** (2021).

66	Duke-Cohan, J. S. *et al.* Attractin (DPPT-L), a member of the CUB family of cell adhesion and guidance proteins, is secreted by activated human T lymphocytes and modulates immune cell interactions. *Proc Natl Acad Sci U S A* **95**, 11336-11341 (1998).

67	Duke-Cohan, J. S., Tang, W. & Schlossman, S. F. Attractin: a cub-family protease involved in T cell-monocyte/macrophage interactions. *Adv Exp Med Biol* **477**, 173-185 (2000).

68	Phillips, R. E., Soshnev, A. A. & Allis, C. D. Epigenomic Reprogramming as a Driver of Malignant Glioma. *Cancer Cell* **38**, 647-660 (2020).

69	Krug, B. *et al.* Pervasive H3K27 Acetylation Leads to ERV Expression and a Therapeutic Vulnerability in H3K27M Gliomas. *Cancer Cell* **35**, 782-797.e788 (2019).

70	Lin, G. L. *et al.* Therapeutic strategies for diffuse midline glioma from high-throughput combination drug screening. *Sci Transl Med* **11** (2019).

71	Mohammad, F. *et al.* EZH2 is a potential therapeutic target for H3K27M-mutant pediatric gliomas. *Nat Med* **23**, 483-492 (2017).

72	Brien, G. L. *et al.* Simultaneous disruption of PRC2 and enhancer function underlies histone H3.3-K27M oncogenic activity in human hindbrain neural stem cells. *Nat Genet* **53**, 1221-1232 (2021).

73	Topper, M. J., Vaz, M., Marrone, K. A., Brahmer, J. R. & Baylin, S. B. The emerging role of epigenetic therapeutics in immuno-oncology. *Nat Rev Clin Oncol* **17**, 75-90 (2020).

74	Reardon, D. A. *et al.* Effect of Nivolumab vs Bevacizumab in Patients With Recurrent Glioblastoma: The CheckMate 143 Phase 3 Randomized Clinical Trial. *JAMA Oncol* **6**, 1003-1010 (2020).

75	Touat, M. *et al.* Mechanisms and therapeutic implications of hypermutation in gliomas. *Nature* **580**, 517-523 (2020).
